# Physiotherapy for patients with hip and knee osteoarthritis in Germany: a survey of current practice

**DOI:** 10.1186/s12891-023-06464-0

**Published:** 2023-05-26

**Authors:** Carolin Bahns, Christian Kopkow

**Affiliations:** Department of Therapy Science I, Brandenburg University of Technology Cottbus-Senftenberg, Universitätsplatz 1, 01968 Senftenberg, Germany

**Keywords:** Osteoarthritis, Physiotherapy, Guideline adherence

## Abstract

**Background:**

Osteoarthritis (OA) of the hip and the knee is common and leads to pain, stiffness and disability. Clinical practice guidelines (CPG) provide recommendations to assist healthcare professionals in clinical decision-making. Although evidence-based physiotherapy has been shown to be effective in the management OA, a gap between clinical practice and guideline recommendations exists. Little is known about OA management provided by physiotherapists in Germany and whether treatment aligns with CPGs. The objectives of this study were (1) to investigate the current physiotherapy practice in patients with hip and/or knee OA in Germany, (2) to evaluate physiotherapists’ adherence to guideline recommendations and (3) to explore barriers and facilitators to guideline use.

**Methods:**

A cross-sectional online survey was conducted among physiotherapists. The questionnaire collected information on demographic characteristics, physiotherapists’ management of hip and knee OA and the use of CPGs in clinical practice. Guideline adherence was evaluated by comparing the survey findings with guideline recommendations. Full adherence was assumed if all recommended treatment options were chosen.

**Results:**

In total, 447 (74.9%) of 597 eligible physiotherapists completed the survey. Data from 442 participants (mean age 41.2 ± 12.8 years; 288 female (65.1%)) were included in the analysis. The most common treatment choices for both hip and knee OA were exercise therapy (hip OA: 424/442, 95.9%; knee OA: 426/442, 96.4%), self-management advice (hip OA: 413/442, 93.2%; knee OA: 395/442, 89.4%) and education (hip OA: 325/442, 73.5%; knee OA: 331/442, 74.9%), followed by manual therapy (hip OA: 311/442, 70.4%; knee OA: 311/442, 70.4%) and joint traction (hip OA: 208/442, 47.1%; knee OA: 199/442, 45.0%). Full guideline adherence was found in 17.2% (76/442) of physiotherapists for hip OA management and in 8.6% (38/442) for knee OA. Less than half of the respondents (212/430, 49.3%) were aware of an OA guideline.

**Conclusions:**

In accordance with current guideline recommendations, most physiotherapists provide exercise therapy and education for patients with hip and/or knee OA. Interventions with low or conflicting evidence were also frequently provided. The limited awareness of existing OA guidelines and the low guideline adherence indicate an insufficient implementation of CPGs in German physiotherapy practice.

**Trial registration:**

German Clinical Trials Register (DRKS00026702). Registered 14 October 2021.

**Supplementary Information:**

The online version contains supplementary material available at 10.1186/s12891-023-06464-0.

## Background

Osteoarthritis (OA) is the most common chronic joint disease and one of the leading causes of disability worldwide [1, 2]. Individuals often suffer from pain, stiffness, functional limitations and reduced quality of life [3]. Although any joint can be affected, OA is mainly present in the large weight-bearing joints such as the hips and knees, as well as in the hands [4–6]. In Germany, the prevalence of hip/knee OA diagnosed by physicians working in outpatient care in elderly patients is 21.8%, whereas OA is more common in women than in men and prevalence increases with age [7]. As a consequence of expanding life expectancy and rising cases of obesity, a further increase of the OA population is expected worldwide and also for Germany [2].

As there is currently no cure for OA, treatment is mainly symptom-based and focuses on pain relief and improving physical function. Clinical practice guidelines (CPG) summarise the available evidence to assist healthcare professionals in clinical decision-making and to optimise patient care [8]. In national [9, 10] and international [11–15] OA guidelines, a conservative non-pharmacological management is recommended and a combination of exercise therapy and patient education (and weight loss) is seen as first-line treatment for patients with hip and knee OA.

However, studies from different countries have shown that physiotherapists also provide a wide range of interventions for patients with hip and/or knee OA [16–26] which do not contribute to high quality of care and are not recommended in CPGs. In a systematic review, Zadro et al. [27] found the median percentage of physiotherapists who chose treatments with low or conflicting evidence in the management of knee OA was 98% (based on n = 5 surveys among physiotherapists). The reasons contributing to the gap between clinical practice and research findings are manifold. Lack of time, patients’ preferences, lack of generalisability of research findings to individual patients and lack of support in the workplace are barriers to guideline use mentioned in literature [28–31]. Furthermore, often physiotherapists are not even aware of current CPGs [32, 33].

In Germany, physiotherapy is prescribed to 43.1% of the patients with hip and/or knee OA diagnosed by physicians working in outpatient care [7], but little is known which interventions are delivered and whether they are in line with guideline recommendations. Although several studies from other countries describing the current physiotherapy practice of patients with hip and/or knee OA exist [16–26], results cannot be transferred to the German context due to differences in the healthcare system, as, for example, direct access has not been established in Germany and physiotherapists are bound to the physician’s prescription.

Identifying gaps between clinical practice and guideline recommendations as well as barriers to guideline use could inform future research and support the development of interventions aiming to implement evidence into OA management to optimise quality of healthcare. Therefore, the objectives of this study were (1) to investigate the current physiotherapy practice in patients with hip and/or knee OA in Germany, (2) to evaluate physiotherapists’ adherence to guideline recommendations and (3) to explore barriers and facilitators to guideline use.

## Methods

### Study design

A cross-sectional study was conducted as a nationwide open online survey. The study was performed in accordance with the Declaration of Helsinki and ethical approval was obtained from the ethics committee of the Brandenburg University of Technology Cottbus-Senftenberg (EK2021-10). The study was prospectively registered on 14 October 2021 with the German Clinical Trials Register (DRKS00026702), which is linked to the International Clinical Trials Registry Platform from the World Health Organization [34]. Reporting of the study followed the Strengthening the Reporting of Observational Studies in Epidemiology (STROBE) guideline for observational studies [35] and the Checklist for Reporting Of Survey Studies (CROSS) [36].

### Participants

Physiotherapists working in Germany, who reported to treat patients with hip or knee OA were included. Participants were recruited through announcements and calls on different physiotherapy networks, articles in newsletters, social media, relevant internet platforms and personal contacts of the study team. The invitation emails and posts contained a short summary about the study content and objective, a link to the online survey and author contact information. Snowball sampling was used and respondents of the questionnaire were encouraged to further distribute the participation invitation. Because this was an explorative study, no power calculation was performed.

### Online survey

The questionnaire (see Additional file 1 for the online survey in German language and Additional file 2 for a translated English language version of the questionnaire) was developed by the authors for the purpose of this study and collected information in three sections:

1. Demographic and occupational characteristics (e.g., sex, age, years of work experience).
2. Physiotherapists’ management of OA: A long list of treatment modalities was developed based on the German guidelines for hip and knee OA [9, 10] and previous studies evaluating current physiotherapeutic care of OA. The frequency of actual use or recommendation of each of the interventions was assessed on a 4-point Likert-type scale (where 1 = never, 2 = sometimes, 3 = mostly, 4 = always), following the methods of previous surveys [17, 20, 21]. Using a closed-ended question, factors that influence the physiotherapists’ treatment choices were recorded.
3. CPGs: In this section, questions were about the awareness of different CPGs for hip and knee OA. Respondents who reported to be aware of the German guidelines were asked about specific barriers and facilitators regarding these guidelines using the translated and cultural adapted version of the “Barriers and Facilitators Assessment Instrument” (BFAI) [37]. Respondents who denied using guideline recommendations in clinical practice were asked about general barriers to guideline use. In the form of a closed-ended question, a list of common barriers described in the literature, such as lack of time, resources or interest in using CPGs, was provided [18, 28–30].

The opening page included written information about the objective, extent, and data storage of the study. Completion of the survey was voluntary, with no incentives offered. Participation was anonymous, and participants had the option of declining to answer specific questions or to leave the questionnaire blank. Before starting the survey, participants gave informed consent by ticking a checkbox to confirm that they had read and understood the study information and agreed to participate in the study. Eligibility criteria were verified by two opening questions asking the participants whether they were currently working as a physiotherapist and whether they treated patients with hip or knee OA. Those who answered “No” to one or both questions were redirected to a “Thank you” page and excluded from the study.

Before dissemination, the survey was pilot-tested with eight physiotherapists (aged between 28 and 38, one of them was male and seven had an academic degree). Using the thinking aloud method, they gave feedback on difficulties in understanding, semantics, conception and layout of the questionnaire. All suggestions obtained in this process were discussed by the authors and minor adjustments were made.

Data collection was conducted between October and December 2021. The questionnaire was administered using LimeSurvey (Hamburg, Germany), a web-based survey design tool. The survey was accessible online without restrictions (password or registration) via an internet link to the survey homepage. No cookie- or IP-based duplicate protection was used to enable multiple participation from shared devices. The questionnaire included 23 items on 11 screens. Through the use of filter questions, respondents were individually navigated through the questionnaire and irrelevant items were eliminated, thus, the number of pages and items per page varied among participants. In case of missing answers, participants were reminded to complete all questions before progress to the next page. Questions were closed or semi-open with single or multiple answers. For all questions, a “no answer” option was available and respondents were allowed to go back to previous pages in the survey. Estimated completion time was approximately 10 min.

### Data analysis

Statistical analysis was performed using the software R Version 3.3.2 (The R Project for Statistical Computing, Vienna, Austria). Using descriptive statistics (e.g., mean values with standard deviation for continuous variables and frequencies/percentages for categorical variables), participants’ characteristics, self-reported current practice and the results regarding CPGs were summarised. Missing data were reported for each variable.

#### Level of guideline adherence

Guideline adherence was defined as the accordance between guideline recommendations and the physiotherapists’ treatment choices. Recommendations provided by the German guidelines for hip and knee OA are provided in Table 1. Interventions with an open recommendation were not included in the analysis. Following the methods described by Battista et al. [19], physiotherapists were classified as ‘Delivering’, ‘Partially Delivering’ and ‘Non-Delivering’. Respondents were considered as ‘Delivering’ if they chose all in the CPGs recommended treatments without selecting any of the non-recommended treatments. They were considered as ‘Partially Delivering’ if they chose at least one of the recommended interventions but none of the non-recommended ones. Those, who chose at least one of the non-recommended treatments and/or none of the recommended ones, were considered as ‘Non-Delivering’. Respondents who gave ‘no answer’ to any of the relevant recommendations were excluded from the analysis.

**Table 1:** Recommendations of the German guidelines for hip and knee OA

	Hip OA [9]	Knee OA [10]
**Recommended**	Exercise therapy	Exercise therapy
		Education
		Weight reduction
	Support for self-management	
	Aquatic exercise	Aquatic exercise
**Not recommended**		Infrared therapy
		Neuromuscular electric stimulation

*OA* Osteoarthritis

In deviation from the study protocol [38], guideline adherence was not evaluated according to the scoring system proposed in the study by Bahns et al. [32]. Since the German guidelines for hip and knee OA only provide few recommendations for or against an intervention, guideline adherence was determined using the methods described by Battista et al. [19] instead of a sum score and a cut-off value of 80% of the maximum score.

#### Determinants of clinical practice

Due to the small sample size of the survey, it was not possible to conduct regression analyses as planned in the study protocol [38]. Instead, bivariate analyses (Chi-square test, Fisher’s Exact Test) were used to determine associations between demographic and occupational characteristics (age, sex, highest professional degree, work experience, work setting, size of city/municipality of employment, awareness of CPGs and number of patients with hip and/or knee osteoarthritis treated per week [16–18, 20]) and the five most common treatment choices. Bonferroni correction was applied to correct for multiple testing [39]. To determine the significance level, a p-value of 0.05 was divided by the number of separate analyses (n = 80, p = 0.000625).

## Results

### Survey response

**Fig. 1:**
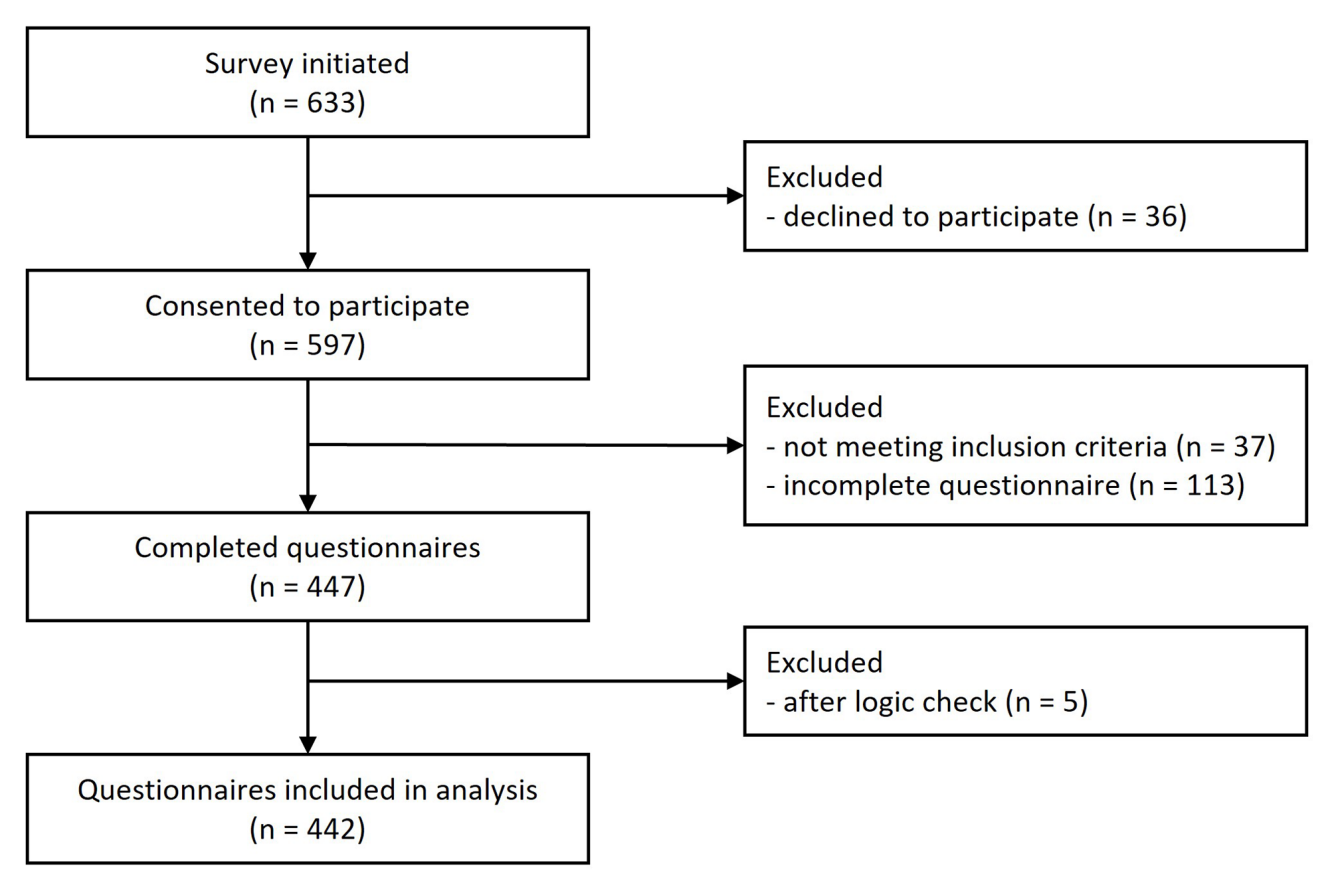
Flow of participants

A total of 633 participants initiated the survey, of whom 597 gave their consent to participate. Among those, 37 were excluded as they did not work as physiotherapists (n = 14), did not treat patients with hip or knee OA (n = 14) or did not meet either of the eligibility criteria (n = 9). Questionnaires were considered ‘complete’, if at least data on physiotherapeutic management of patients with hip and knee OA was provided. After a plausibility check, five questionnaires were excluded, leaving a final sample of 442. The completion rate (completers/participants) was 78.9%. The study participation process is displayed in Fig. 1.

### Characteristics of the study population

From the 442 physiotherapists who were included in analysis, 288 (65.2%) were women and 152 (34.4%) men. The mean age was 41.2 years (SD 12.8), the mean work experience was 17.3 years (SD 12.3). Most respondents were primarily working in a private practice (378/442, 85.5%) and had graduated from vocational school (298/442, 67.4%). Further demographic and occupational characteristics are summarised in Table 2. A distribution of the respondents across the 16 German federal states is displayed in Additional file 3 (Figure A).

**Table 2:** Characteristics of participants (N = 442)

Characteristic	
Age in years, mean (SD) range	41.2 (12.8) 21–70 *(n = 440)*
Sex, n (%)	
Female	288 (65.2)
Male	152 (34.4)
Diverse	1 (< 1)
No answer	1 (< 1)
Highest professional degree, n (%)	
Diploma (vocational school)	298 (67.4)
Bachelor/diploma (university)	106 (24.0)
Master	28 (6.3)
Doctorate	0 (0)
No answer	9 (2.0)
Missing	1 (< 1)
Work experience in years, mean (SD) range	17.3 (12.3) 0–55 *(n = 442)*
Work setting, n (%)	
Private practice	378 (85.5)
Hospital	28 (6.3)
Rehabilitation clinic	29 (6.6)
Other	5 (1.1)
No answer	0 (0)
Missing	2 (< 1)
Size of the city/municipality of employment, n (%)	
Rural area (< 5.000 inhabitants)	64 (14.5)
Small town (5.000–20.000 inhabitants)	124 (28.1)
Mid-sized town (20.000–100.000 inhabitants)	114 (25.8)
Large town (> 100.000 inhabitants)	131 (29.6)
No answer	9 (2.0)
Number of working hours per week, median (25th -75th percentile) range	35.5 (28.3–40.0) 5–65 *(n = 442)*
Number of hip and/or knee OA patients seen per week, median (25th -75th percentile) range	6 (4–10) 1–40 *(n = 440)*

*OA* Osteoarthritis; *SD* Standard Deviation. Note: Different sample size within each sample due to missing values

### Current clinical practice

**Fig. 2:**
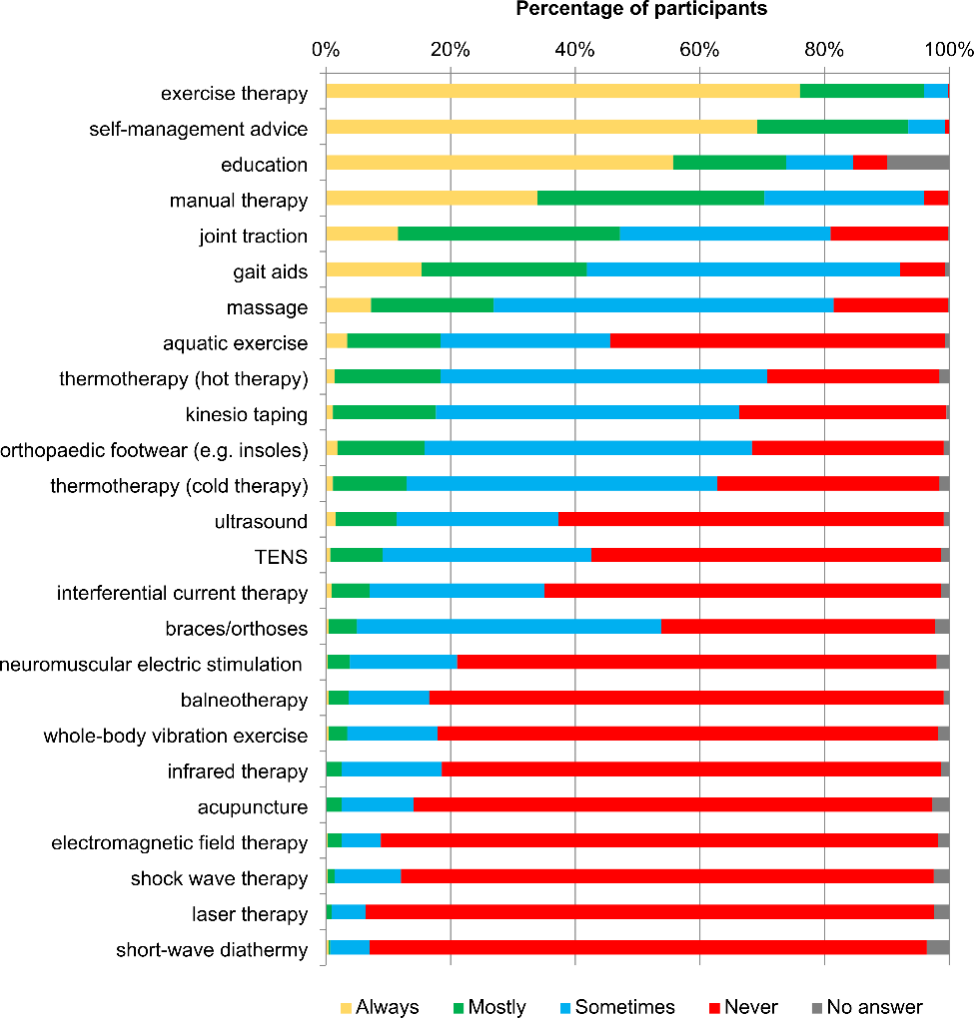
Reported physiotherapy interventions when treating hip osteoarthritis *TENS* Transcutaneous electrical nerve stimulation

**Fig. 3:**
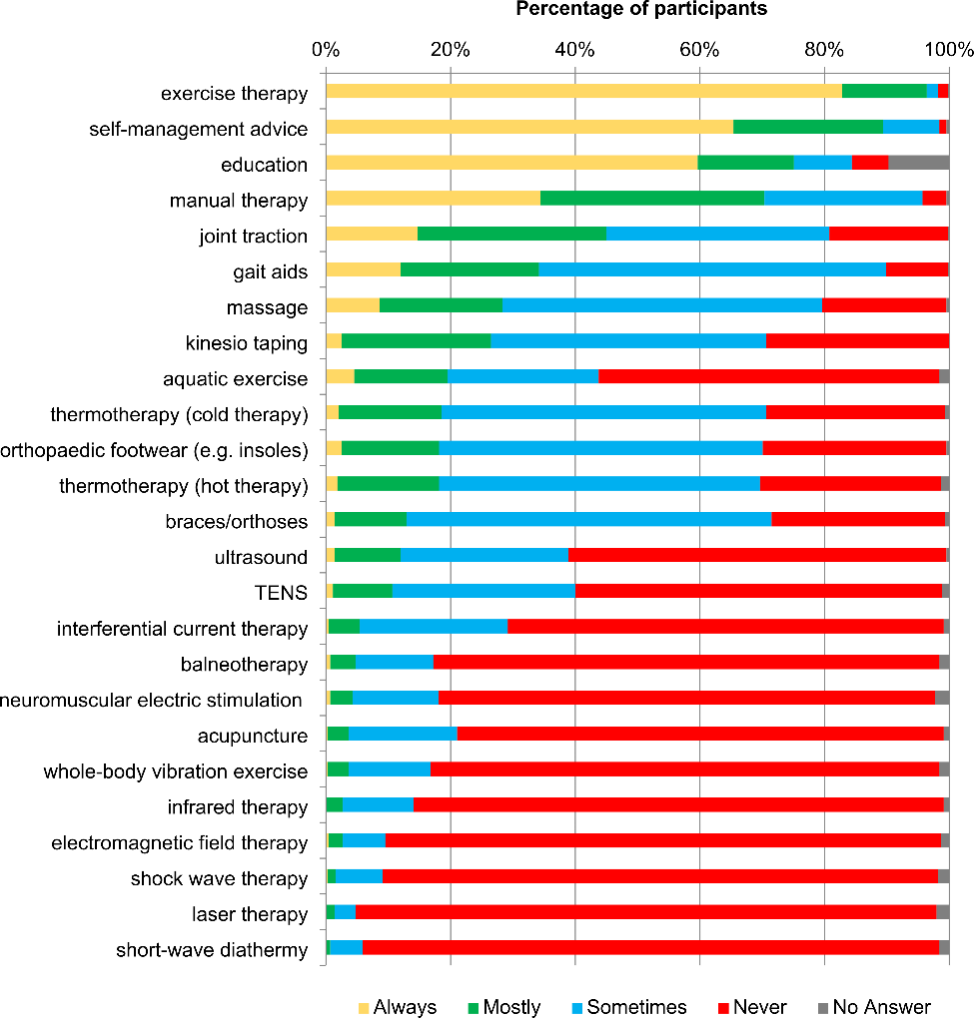
Reported physiotherapy interventions when treating knee osteoarthritis *TENS* Transcutaneous electrical nerve stimulation

For both hip and knee OA, the five most common treatment choices were exercise therapy (hip OA: 424/442, 95.9%; knee OA: 426/442, 96.4%), self-management advice (hip OA: 413/442, 93.2%; knee OA: 395/442, 89.4%), education (hip OA: 325/442, 73.5%; knee OA: 331/442, 74.9%), manual therapy (hip OA: 311/442, 70.4%; knee OA: 311/442, 70.4%) and joint traction (hip OA: 208/442, 47.1%; knee OA: 199/442, 45.0%). A full list of reported treatment choices is displayed in Figs. 2 and 3 (for absolute numbers see Additional file 3, Table A and B).

Regarding patient education, over 90% of the respondents reported to discuss pain science (hip OA: 310/325, 95.4%; knee OA: 308/331, 93.1%), exercise dose (hip OA: 307/324, 96.8%; knee OA: 304/331, 91.8%) and the benefits of a healthy lifestyle (hip OA: 293/324, 90.2%; knee OA: 299/331, 90.3%). Weight reduction was mentioned least often by the respondents (hip OA: 206/325, 63.4%; knee OA: 222/331, 67.1%). Of those who reported providing self-management advice, advice on pain management was most common (hip OA: 377/413, 91.3%; knee OA: 358/393, 91.1%). Details on patient education and self-management advice are given in Additional file 3 (Table A and B).

The results of the Chi-square/Fisher’s test analyses (see Additional file 3, Table C and Table D) showed that there was a significant association between choosing exercise therapy for hip OA and the awareness of CPGs. Providing patient education was significantly associated with the highest professional degree and the awareness of CPGs for hip OA, and as well with the awareness of CPGs for knee OA. Choosing manual therapy as a treatment was associated with age, work experience and the awareness of CPGs for both hip and knee OA. A significant association between using manual traction and gender was found for knee OA. No significant associations were found for self-management advice.

The median number of treatment modalities chosen was 5 (range 1–17) for both hip and knee OA. The most commonly used combination of treatments was exercise therapy, self-management advice and education (hip OA: 58/442, 13.1%; knee OA: 60/442, 13.6%), followed by the combination of these three interventions plus the use of manual therapy (hip OA: 20/442, 4.5%; knee OA: 21/442, 4.8%) (see Additional file 3, Table E).

When choosing treatment options for patients with hip and/or knee OA, most participants consider their own clinical experience (355/438, 81.1%) and what they have learned in postgraduate courses (351/438, 80.1%). Recommendations from CPGs seemed to be less important in clinical decision-making (209/438, 47.7%). A full list of factors influencing treatment decisions is displayed in Additional fil 3 (Figure B).

### Guideline adherence

Full guideline adherence in the management of hip OA was found in 17.2% (76/442) of the respondents. 81.2% (359/442) were classified as ‘Partially Delivering’ and 0.7% (3/442) as ‘Non-Delivering’. For knee OA, 8.6% (38/442) of physiotherapists provided all the interventions recommended by the CPG without selecting one of those not recommended. 72.2% (319/442) were classified as ‘Partially Delivering’ and 6.1% (27/442) as ‘Non-Delivering’.

### Awareness of CPGs and perceived barriers

Almost half of the respondents (212/430, 49.3%) reported they were aware of CPGs for hip and/or knee OA. The most commonly named guidelines were the national guidelines for hip (160/212, 75.5%) [9] and knee OA (162/212, 76.4%) [10], followed by the Dutch guideline for hip and knee OA published by the Royal Dutch Society for Physical Therapy (KNGF, Koninklijk Nederlands Genootschap voor Fysiotherapie) (43/212, 20.3%) [13]. Of those therapists who were not aware of a CPG, 134/163 (82.2%) indicated general interest in incorporating guideline recommendations into their future clinical practice.

Physiotherapists who stated not using guidelines in clinical practice most often criticise that guideline recommendations are too unspecific and do not address the individuality of their patients (unspecific: 49/140, 35%; OA: 38/74, 51.4%) (Table 3). A full list of barriers to guideline use is displayed in Table 3.

**Table 3:** Barriers against guideline use

	Unspecific^1^ (n = 140)	OA^2^ (n = 74)
I do not know any guidelines.	38 (27.1)	-
I do not have time to read guidelines.	47 (33.6)	19 (25.7)
I do not have time to implement guideline recommendations in clinical practice.	17 (12.1)	14 (18.9)
I have no interest in using guideline recommendations in clinical practice.	3 (2.1)	2 (2.7)
I do not know how and where to find guidelines.	42 (30.0)	2 (2.7)
There are no/too few guidelines.	12 (8.6)	6 (8.1)
The guideline recommendations are too unspecific and do not address the individuality of my patients.	49 (35.0)	38 (51.3)
Guideline recommendations are not helpful to improve patient care.	8 (5.7)	5 (6.8)
My colleagues do not support the use of guidelines.	10 (7.1)	14 (18.9)
My supervisor does not support the use of guidelines.	18 (12.9)	15 (20.3)
Guideline recommendations contradict my clinical expertise.	3 (2.1)	2 (2.7)
Guideline recommendations hinder me in my clinical decision-making.	7 (5.0)	3 (4.1)
My patients’ preferences do not match the guideline recommendations.	15 (10.7)	24 (32.4)
OA of the hip/knee joints is not a serious disease and does not require management according to guidelines.	-	1 (1.3)
I have difficulties in understanding and critically appraising guidelines.	8 (5.7)	1 (1.3)
I do not have the resources (e.g. rooms, equipment) to implement guideline recommendations in clinical practice.	15 (10.7)	10 (13.5)
Others	7 (5.0)	5 (6.8)
No answer	10 (7.1)	11 (14.9)

^1^Physiotherapists, who reported not to use any guideline in clinical practice

^2^Physiotherapists, who reported not to use OA guidelines in clinical practice

*OA* Osteoarthritis

Explicitly in relation to the German guidelines for hip [9] and knee OA [10], respondents agreed that the guideline was a good starting point for their self-study (119/143, 83.2%), leaving enough room to make own conclusions (120/143, 83.9%) and to weigh their patients’ wishes (109/143, 76.2%). In line with the barriers mentioned in Table 3, colleagues (58/143, 40.6%), other healthcare professionals (75/143, 52.4%) and patients (46/143, 32.2%) unwilling to follow the guideline are barriers in translating evidence-based recommendations into clinical practice (see Additional file 3, Table F).

## Discussion

A nationwide online survey was conducted to assess current clinical practice in patients with OA and to evaluate physiotherapists’ adherence to guideline recommendations as well as barriers and facilitators to guideline use. The findings of the study indicate that for both hip and knee OA the most commonly used or advised physiotherapy interventions are exercise therapy, self-management advice and education. This is in line with national and international guidelines [9–14]. However, German physiotherapists also frequently provide interventions with low or conflicting evidence e.g., manual therapy or joint traction. Since most respondents did not consider all interventions recommended in the German guidelines for OA management, overall adherence was low and less than half of the respondents were aware of an OA guideline.

The findings regarding clinical practice are comparable to recent findings from Italy [19], Australia [40], Canada [18], Belgium [20] and the UK [24], showing that the majority of therapists provide or advise exercise therapy, education and/or self-management advice for patients with hip and knee OA. However, although weight management for obese or overweight individuals with OA (especially for knee OA) is also considered a first-line treatment in CPGs, German physiotherapists are least likely to address this aspect as part of patient education. This may be because weight management conversation is perceived as potentially sensitive and patients may feel stigmatised by discussions about their weight. In addition, some physiotherapists may consider weight management to be outside their clinical scope and physiotherapy skills. Findings from other studies have shown that physiotherapists feel they lack knowledge and confidence to appropriately initiate and support weight loss [40–42].

Consistent with the findings of other studies [16, 19, 20, 24], German physiotherapists frequently use interventions with uncertain or low-level evidence in addition to those recommended in guidelines. For both hip and knee OA, manual therapy is ‘always’ or ‘mostly’ used by up to 70% of respondents. Joint traction, massage and, for knee OA, kinesio taping are also provided by more than 20% of physiotherapists. In this survey, most respondents indicated that their choice of physiotherapy interventions was primarily influenced by their own clinical experience and by content learned in postgraduate courses, whereas evidence from current research and recommendations from OA guidelines appeared to be less important. Possible explanations for why physiotherapists frequently use interventions with little or unknown value may include the need for clinical innovation and the increased exposure to treatments of unknown value via social media or postgraduate courses [43]. At the same time, keeping up to date with evidence is challenging [43]. Many physiotherapists report lack of research skills, such as critically appraising evidence from literature. However, lack of time is considered the most important barrier to staying up-to-date, as searching databases and interpreting research findings is time-consuming and the body of evidence in physiotherapy research is growing [29, 44].

Less than half of the respondents (212/430, 49.3%) were aware of an OA guideline. This is comparable to the results of other surveys among German physiotherapists, where only 29% were aware of a CPG for low back pain [32] and 52.9% were aware of a CPG for stroke management [33]. As there is a significant association between guideline awareness and the choice of physiotherapy interventions, improved implementation strategies appear reasonable.

Guideline adherence in German physiotherapists seems to be low, and in comparison to the study of Battista et al. [19] large differences can be observed: 25% of the Italian physiotherapists were classified ‘Delivering’, 22% as ‘Partially Delivering’ and 53% as ‘Non-Delivering’. This could be due to the fact that less than half of the German physiotherapists reported to be aware of an OA guideline with even fewer therapists stated using those CPGs in clinical practice. Another explanation could be that while in Italy a bachelor’s degree is required as minimum qualification to practice [45], only about 3% of German physiotherapists are graduated from higher educational institutions [46]. Most physiotherapists are trained at vocational schools according to the German Training and Examination Regulations for Physiotherapists, which does not address special competencies regarding the implementation of guidelines and evidence-based practice [47]. However, Husted et al. [48] have also reported variations in guideline adherence across different countries. To date, there is no standardised method for measuring guideline adherence and different approaches, such as questionnaires (case vignettes [19], self-report or practice [20]), direct observations [49] or chart audits [50] are used, limiting comparison of results. A consensus on how to define and assess guideline adherence would be helpful to better compare clinical practice in future studies. Another important factor that might influence findings on guideline adherence across studies is the guideline itself and the interventions considered. Although national and international guidelines agree in exercise therapy and education as first-line treatments for OA, recommendations for other physiotherapeutic interventions vary.

The findings of this study indicate an insufficient implementation of OA guidelines in German physiotherapy. Based on the results, the publication and dissemination of guidelines alone is not sufficient to change clinical practice, as 38.9% (74/190) of physiotherapists reported not to consider guideline recommendations even though they were aware of an OA guideline. In line with previous research [29], respondents to the survey mentioned different contextual factors that hinder guideline use in clinical practice, such as lack of time to read or implement guideline recommendations and lack of resources/equipment. Consistent with research specific to evidence-based practice [44], the results confirm that German physiotherapists perceive guideline recommendations too unspecific and that they do not address the individuality of their patients (38/74, 51.3%). In addition, as also mentioned in other studies [28, 29], disagreement between recommendations and patient expectations as well as lacking support from supervisors and colleagues were reported as important barriers. However, the majority of physiotherapists who were not aware of any OA guideline at the time of the survey stated that they were interested in using guideline recommendations in future, indicating a positive attitude towards CPGs. Most respondents considered the German OA guidelines a good starting point for their self-study and believed that the guideline leaves enough room for their own conclusions. Tailored implementation strategies are needed that overcome contextual barriers while strengthening existing facilitators and aim to change physiotherapists’ behaviour and attitudes towards evidence-based guidelines.

Currently, there are already several approaches to implement evidence-based physiotherapy for individuals with OA. For example, Good Life with osteoArthritis in Denmark (GLA:D®) has been shown to be effective in reducing pain and increasing quality of life and physical function [51]. GLA:D® has been implemented in several countries, e.g., Canada, Australia and New Zealand, and is currently also being implemented in Germany. However, data from Australia show that implementing new pragmatic approaches takes time: Only 7% of all musculoskeletal physiotherapists were reached with GLA:D® Australia training over a three-year period, leaving much room for further implementation [52]. In addition, since not all physiotherapists want to be trained in GLA:D®, as it requires a two-day paid training course and entry of patient data into a national registry is mandatory, and not all patients can or want to participate in a group program, it is necessary to further evaluate how to increase guideline use in OA care.

### Limitations

Although much effort was made to distribute the survey broadly, it seems that the majority of physiotherapists working in Germany were not aware of the study, as the number of participants (n = 442) was low compared to the total number of approximately 203,000 physiotherapists working in Germany [46].

As common to all surveys, sampling and volunteer biases may limit the generalisability of the study findings to the wider population of German physiotherapists. Using an online survey may have introduced bias by mainly including (younger) physiotherapists familiar with the use of digital media. It is also likely that physiotherapists with a personal interest in hip or knee OA may have participated disproportionately and might have better knowledge of OA management. Moreover, participants with an academic background (30.3%) were overrepresented in the study sample compared to their assumed number in Germany (3%) [46]. The analyses showed that there is an association between the highest professional degree and the choice of different treatment modalities. As those have more knowledge about evidence-based practice, the findings of this study might overestimate the real level of current physiotherapy practice and adherence to OA guidelines.

The study findings do not allow to distinguish what the physiotherapists do or what they recommend to do. In addition, the current data are based on physiotherapists self-report and may not reflect what kind of care therapists actually provide in clinical practice. Due to social desirability, participants may have over-reported recommended behaviours and under-reported behaviour contrary to guideline recommendations. However, the results of a recent study assessing current physiotherapy practice through clinical audits are comparable to results obtained through self-report [26]. By combining the current survey data with data from e.g., patient surveys, chart audits or clinical observations, a more reliable picture of OA management in physiotherapy could be established.

Finally, although clinical practice seems to be largely in line with guideline recommendations, no conclusions can be drawn on the quality of OA management. The type of exercises was not specified, and it is therefore unclear whether physiotherapist provide e.g., endurance training, strength training or neuromuscular exercises that have shown to be safe and effective in patients with OA [51]. In addition, the frequency, duration or intensity of each intervention provided were not recorded.

## Conclusions

Physiotherapists in Germany commonly provide or advise interventions for patients with hip and/or knee OA that are in line with current guideline recommendations, including exercise therapy and education. However, they also reported to use a variety of interventions with low or conflicting evidence. Differences in clinical practice have been observed depending on physiotherapist’s highest educational degree and the awareness of guidelines. The low guideline adherence and the limited awareness of existing OA guidelines indicate an insufficient implementation of CPGs in German physiotherapy practice. A tailored implementation intervention addressing the most important barriers against guideline use may optimise clinical practice and result in improved patient care.

### Electronic supplementary material

Below is the link to the electronic supplementary material.

Supplementary Material 1

Supplementary Material 2

Supplementary Material 3

## Data Availability

The datasets generated during the current study and R syntax for data preparation and analysis are available on the Open Science Framework: 10.17605/OSF.IO/A7P3W.

## List of abbreviations

BFAI: Barriers and Facilitators Assessment Instrument
CPG: clinical practice guideline
GLA:D®: Good Life with osteoArthritis in Denmark
OA: osteoarthritis
TENS: transcutaneous electrical nerve stimulation
