# Supplementary material for: Physiotherapy for patients with hip and knee osteoarthritis in Germany: a survey of current practice
Source: BMC Musculoskelet Disord. 2023 May 26;24:424. doi: 10.1186/s12891-023-06464-0 (PMC10262543; doi:10.1186/s12891-023-06464-0)
Supplement: Supplementary file 1 — Supplementary Material 1 [file 12891_2023_6464_MOESM1_ESM.pdf]

Additional file 2: Translated English language version of the questionnaire

Please note: This English language version is a direct translation of the original questionnaire without any form of cultural adaptation.

Physiotherapy for patients with hip and/or knee osteoarthritis in Germany

Dear Participants,

Thank you very much for your interest in our online survey on “Physiotherapy for patients with hip and/or knee osteoarthritis in Germany.”

What is the aim of the study?
The aim of the study is to evaluate the current physiotherapy management of patients with hip and/or knee osteoarthritis in Germany. We would like to investigate to what extent clinical practice is in accordance with the recommendations of the German AWMF guidelines for hip and knee osteoarthritis. In addition, we would like to know which factors influence the implementation and use of guideline recommendations in physiotherapy practice.

What is the study about?
We would like to ask you a few questions about the following subject areas:

1. Demographic and occupational characteristics
2. Physiotherapy management of hip and knee osteoarthritis
3. Barriers and facilitators to guideline use

How much time does it take to participate?
Participation in the online survey takes about <u>10 minutes</u>.

What happens with my data?
Obtained data will be used exclusively for scientific purposes. Commercial use is excluded. Only anonymised data will be used for analysis, i.e. information cannot be linked to a specific person. The data will be stored on a separate server at the Brandenburg University of Technology Cottbus-Senftenberg for at least 10 years after the end of the study. The dataset will be made available to the research community in a secure, internet-based data archive (Open Science Framework). Thus, the study follows the recommendations of the German Research Foundation (DFG) for quality assurance in research.

What are the risks?
The survey is not associated with any risks.

Your participation in this study is voluntary. If you decide to take part, you are still free to withdraw yourself and your data from the study without giving a reason. You are not obligated to answer all questions and you can stop the survey at any time.

If you have any further questions about the study or if you have not understood anything in this participant information, please contact Prof. Dr. Christian Kopkow:

Phone: 03573 - 85741

Email: christian.kopkow@b-tu.de

Data Privacy Statement

- I hereby grant my consent for the data gathered in this study to be recorded and stored in an anonymised form at the study centre* for scientific purposes. I further agree that the data will be made accessible to the research community in a secure, internet-based data archive following the recommendations of the German Research Foundation for quality assurance in research.
- I also agree that an authorised member of the ethics committee who is bound to secrecy may have access to my personal data held by the study centre*, insofar as this is necessary for the validation of the study. In this case, I release the study centre from its duty of confidentiality.
- I understand that I can withdraw from the study at any time without facing any negative consequences.

* Prof. Dr Christian Kopkow, Brandenburg University of Technology, Department of Therapy Science I, Universitätsplatz 1, 01968 Senftenberg

I have understood the participant information on the study "Physiotherapy for patients with hip and/or knee osteoarthritis in Germany" and I consent to participate in the study and to the anonymised recording and analysis of my data, the transfer the data to others and the examination of my personal data in the form described above.

Click **"Continue"** to participate in the study.

Please answer the following questions to verify your eligibility to participate in this study:

1. Do you currently work as a physiotherapist in Germany?

   ○ Yes

   ○ No

2. Does your current work involve the management of patients with hip and/or knee osteoarthritis?

   ○ Yes

   ○ No

I. Demographic and occupational characteristics

3. What is your age?

   [Please select] ○ No answer

4. What is your gender?

   ○ Male

   ○ Female

   ○ Divers

   ○ No answer

5. In what federal state of Germany do you work?

   [Please select] ○ No answer

6. How many inhabitants live in the town (or municipality) of your workplace?

   ?? *If you have more than one place of work, please answer this question with regard to the workplace where you work most of the time.*

   ○ < 5,000 inhabitants (rural area)

   ○ 5,000 - 20,000 inhabitants (small town)

   ○ 20,000 - 100,000 inhabitants (mid-sized town)

   ○ > 100,000 inhabitants (large town)

   ○ No answer

7. How many years of work experience do you have as a physiotherapist?

   [Please select]   ○ No answer

8. How many hours per week do you typically work as a physiotherapist?

   [Please select]   ○ No answer

9. In what type of setting do you work?

   ?? If you have more than one place of work, please answer this question with regard to the workplace where you work most of the time.

   ○ Private practice

   ○ Hospital

   ○ Rehabilitation clinic

   ○ Other: _____

   ○ No answer

10. What is your highest level of physiotherapy education completed (or comparable field, e.g. health sciences, public health, sport science)?

   ○ Diploma (vocational school)

   ○ Diploma (university)

   ○ Bachelor

   ○ Master

   ○ Doctorate

   ○ Other: _____

   ○ No answer

11. How many patients with hip and/or knee osteoarthritis do you currently treat on average in a typical working week?

   ?? Patients who are treated several times a week only count once.

   [Please select]   ○ No answer

Now we would like to learn more about the physiotherapy management of patients with hip and knee osteoarthritis.

Please note that there are two separate tables. The first table refers explicitly only to the treatment of hip osteoarthritis, in a second table you will subsequently be asked about the treatment of knee osteoarthritis.

II. Physiotherapy management of hip and knee osteoarthritis

12. Please indicate how often you use or recommend the following interventions in the management of patients with **hip osteoarthritis**.

    ?? *The interventions are listed in alphabetical order.*

	Never	Sometimes	Mostly	Always	No answer
Acupuncture	o	o	o	o	o
Balneotherapy	o	o	o	o	o
Exercise therapy	o	o	o	o	o
Education	o	o	o	o	o
➢ Pathology and risk factors	o	o	o	o	o
➢ Impact on function, activity, participation	o	o	o	o	o
➢ Joint protection strategies	o	o	o	o	o
➢ Weight reduction	o	o	o	o	o
➢ Benefits of a healthy lifestyle	o	o	o	o	o
➢ Pain science	o	o	o	o	o
➢ Exercise dose	o	o	o	o	o
Self-management advice	o	o	o	o	o
➢ Pain management advice	o	o	o	o	o
➢ Stress management	o	o	o	o	o
Gait aids	o	o	o	o	o
Whole-body vibration exercise	o	o	o	o	o
Aquatic exercise	o	o	o	o	o
Infrared therapy	o	o	o	o	o
Interferential current therapy	o	o	o	o	o
Electromagnetic field therapy	o	o	o	o	o
Manual therapy	o	o	o	o	o
Massage	o	o	o	o	o
Neuromuscular electric stimulation	o	o	o	o	o
Braces/orthoses	o	o	o	o	o
Orthopaedic footwear (e.g. insoles)	o	o	o	o	o
Shock wave therapy	o	o	o	o	o
Thermotherapy (cold therapy)	o	o	o	o	o
Thermotherapy (hot therapy)	o	o	o	o	o

Joint traction					
Transcutaneous electrical nerve stimulation (TENS)	o	o	o	o	o
Ultrasound	o	o	o	o	o

13. Please indicate how often you use or recommend the following interventions in the management of patients with **knee osteoarthritis**.

    ?? The interventions are listed in alphabetical order.

	Never	Sometimes	Mostly	Always	No answer
Acupuncture	o	o	o	o	o
Balneotherapy	o	o	o	o	o
Exercise therapy	o	o	o	o	o
Education	o	o	o	o	o
➢ Pathology and risk factors	o	o	o	o	o
➢ Impact on function, activity, participation	o	o	o	o	o
➢ Joint protection strategies	o	o	o	o	o
➢ Weight reduction	o	o	o	o	o
➢ Benefits of a healthy lifestyle	o	o	o	o	o
➢ Pain science	o	o	o	o	o
➢ Exercise dose	o	o	o	o	o
Self-management advice	o	o	o	o	o
➢ Pain management advice	o	o	o	o	o
➢ Stress management	o	o	o	o	o
Gait aids	o	o	o	o	o
Whole-body vibration exercise	o	o	o	o	o
Aquatic exercise	o	o	o	o	o
Infrared therapy	o	o	o	o	o
Interferential current therapy	o	o	o	o	o
Electromagnetic field therapy	o	o	o	o	o
Manual therapy	o	o	o	o	o
Massage	o	o	o	o	o
Neuromuscular electric stimulation	o	o	o	o	o
Braces/orthoses	o	o	o	o	o
Orthopaedic footwear (e.g. insoles)	o	o	o	o	o
Shock wave therapy	o	o	o	o	o
Thermotherapy (cold therapy)	o	o	o	o	o
Thermotherapy (hot therapy)	o	o	o	o	o
Joint traction					
Transcutaneous electrical nerve stimulation (TENS)	o	o	o	o	o
Ultrasound	o	o	o	o	o

14. Regarding your answers to the previous questions – what factors influence your choice of treatment options?

    ?? Multiple answers possible

    o Own clinical experience

    o Work setting (e.g. equipment and space)

    o Postgraduate physiotherapy courses

    o Recommendations from osteoarthritis guidelines

    o Content, I learned during my vocational training or at university

    o Colleagues or employees

    o Preferences of my patients

    o Time

    o Current research

    o Other: _____

    o No answer

III. Barriers and facilitators to guideline use

15. Are you aware of guidelines for the management of patients with hip and/or knee osteoarthritis?

    o Yes

    o No

    o No answer

(Note: Questions 16 and 17 only appear if question 15 was answered with "Yes".)

16. Which of the following guidelines are you aware of?

    ?? Multiple answers possible

    □ **AAOS** – Clinical Practice Guideline on the Management of Osteoarthritis of the Hip

    □ **AAOS** – Clinical Practice Guideline on Treatment of Osteoarthritis of the Knee

    □ **ACR** – Guideline for the Management of Osteoarthritis of the Hand, Hip and Knee

    □ **APTA** – Hip Pain and Mobility Deficits – Hip Osteoarthritis: Revision 2017

    □ **AWMF** – S2k-Leitline "Gonarthrose"

    □ **AWMF** – S2k-Leitlinie "Koxarthrose"

    □ **KNGF** – Guideline for Physical Therapy in patients with Osteoarthritis of the hip and knee

□ **EULAR** – Recommendations for the non-pharmacological core management of hip and knee osteoarthritis

□ **NICE** – Osteoarthritis: Care and Management in Adults

□ **OARSI** – Guidelines for the non-surgical management of knee, hip and polyarticular osteoarthritis

□ **RACGP** – Guideline for the management of knee and hip osteoarthritis

□ No answer

17. How do you know these guidelines?

    ?? Multiple answers possible

    □ Vocational school/university

    □ Postgraduate physiotherapy courses

    □ Colleagues/employees

    □ Congresses

    □ Webinars/Podcasts

    □ Social media (e.g. Facebook, Twitter, Instagram, YouTube)

    □ Online (Google, guideline websites)

    □ Professional journals for medicine/health/physiotherapy

    □ Other: _____

    □ No answer

(Note: Question 18 only appears if question 16 was answered with "AWMF – S2k-Leitlinie Gonarthrose" and/or "AWMF – S2k-Leitlinie Koxarthrose".)

18. You have responded that you are aware of the **AWMF S2k-Leitlinien "Koxarthrose" and/or "Gonarthrose"**.
    Following are a couple of statements about working according to these guidelines. We would like to know whether you agree with the statement or not and in what degree. If you do not have a strong opinion, please try to find out if it is more like 'agree' or more like 'disagree'. If you really do not know, you can select the option 'do not agree nor disagree'

	Fully disagree	Disagree	Do not agree nor disagree	Agree	Fully agree	No answer
The guideline leaves enough room for me to make my own conclusions.						
The guideline leaves enough room to weight the wishes of the patient.						

The guideline is a good starting point for my self-study.						
I did not thoroughly read nor remember the guideline.						
I wish to know more about the guideline before I decide to apply it.						
I have problems changing my old routines.						
I think parts of the guideline are incorrect.						
I have a general resistance to working according protocols.						
My colleagues do not cooperate in applying the guideline.						
Other health care professionals do not cooperate in applying the guideline.						
Managers/directors do not cooperate in applying the guideline.						
Patients do not cooperate applying the guideline.						
Working to the guideline is too time consuming.						
The guideline does not fit into my ways of working in practice.						
Working according to this guideline requires financial compensation.						
The layout of the guideline makes it handy for use.						

(Note: Question 19 only appears if question 15 was answered with "Yes".)

19. How often do you use guidelines to inform your treatment of hip and/or knee osteoarthritis?

o Always

o Mostly

o Sometimes

o Never

o No answer

(Note: Question 29 only appears if question 19 was answered with "Sometimes" or "Never".)

20. For what reasons?

?? Multiple answers possible

□ I do not have time to read guidelines

□ I do not have time to implement guideline recommendations at work

□ I have no interest in implementing guideline recommendations at work

□ I do not know how and where to find guidelines

□ There are no/too few guidelines

□ Guideline recommendations are too unspecific and are not suitable given the individuality of the patients

□ Guideline recommendations are not helpful to improve quality of care

□ My colleagues do not support the implementation of guidelines

□ My employer does not support the implementation of guidelines

□ Guideline recommendations contradict my own clinical expertise

□ Guideline recommendations hinder me in clinical decision-making

□ My patients' preferences do not match the guideline recommendations

□ Hip and knee osteoarthritis is not a serious condition and does not require guideline-based treatment

□ I find it difficult to understand and critically evaluate guidelines

□ I do not have the resources (e.g. equipment, space) to implement guideline recommendations in clinical practice

□ Other: ______

□ No answer

(Note: Question 21 only appears if question 15 was answered with "No".)

21. Do you have a general interest in considering recommendations from guidelines in your daily work with patients suffering from hip and/or knee osteoarthritis?

o Yes

o No

o No answer

(Note: Question 22 only appears if question 15 was answered with "No".)

22. How often do you generally use guidelines to inform your clinical practice?

o Always

o Mostly

o Sometimes

o Never

o No answer

(Note: Question 23 only appears if question 22 was answered with "Sometimes" or "Never".)

23. For what reasons?

?? *Multiple answers possible*

□ I am not aware of any guideline

□ I do not have time to read guidelines

□ I do not have time to implement guideline recommendations at work

□ I have no interest in implementing guideline recommendations at work

□ I do not know how and where to find guidelines

□ There are no/too few guidelines

□ Guideline recommendations are too unspecific and are not suitable given the individuality of the patients

□ Guideline recommendations are not helpful to improve quality of care

□ My colleagues do not support the implementation of guidelines

□ My employer does not support the implementation of guidelines

□ Guideline recommendations contradict my own clinical expertise

□ Guideline recommendations hinder me in clinical decision-making

□ My patients' preferences do not match the guideline recommendations

□ I find it difficult to understand and critically appraise guidelines

□ I do not have the resources (e.g. equipment, space) to implement guideline recommendations in clinical practice

□ Other: ______

□ No answer

Thank you very much for your participation!

We would like to thank you very much for your help.

Your answers have been saved and you can now close the browser window.

(Note: This information only appears if question 1 or 2 was answered with "No".)

Thank you very much for your interest in our study!

Unfortunately, you do not meet all the eligibility criteria to participate in the survey.
