# Supplementary material for: Physiotherapy for patients with hip and knee osteoarthritis in Germany: a survey of current practice
Source: BMC Musculoskelet Disord. 2023 May 26;24:424. doi: 10.1186/s12891-023-06464-0 (PMC10262543; doi:10.1186/s12891-023-06464-0)
Supplement: Supplementary file 2 — Supplementary Material 2 [file 12891_2023_6464_MOESM2_ESM.pdf]

**Additional file 3:** Additional results

Figure A: Distribution of the respondents across the 16 German federal states

| German federal state | Physiotherapists | n | Percent |
|---|---|---|---|
| Saarland | 1,258 | 19 | 1.51 |
| Rhineland-Palatinate | 6,242 | 77 | 1.23 |
| Bremen | 958 | 12 | 1.25 |
| Brandenburg | 4,291 | 17 | 0.40 |
| Hamburg | 3,141 | 12 | 0.38 |
| Schleswig-Holstein | 4,732 | 16 | 0.34 |
| Baden-Württemberg | 15,981 | 52 | 0.33 |
| Hesse | 8,457 | 28 | 0.33 |
| North Rhine-Westphalia | 25,515 | 75 | 0.29 |
| Bavaria | 19,605 | 57 | 0.29 |
| Thuringia | 3,866 | 10 | 0.26 |
| Saxony-Anhalt | 4,046 | 7 | 0.25 |
| Lower Saxony | 12,647 | 27 | 0.21 |
| Saxony | 9,433 | 19 | 0.20 |
| Mecklenburg-Vorpommern | 2,676 | 5 | 0.19 |
| Berlin | 6,462 | 9 | 0.14 |

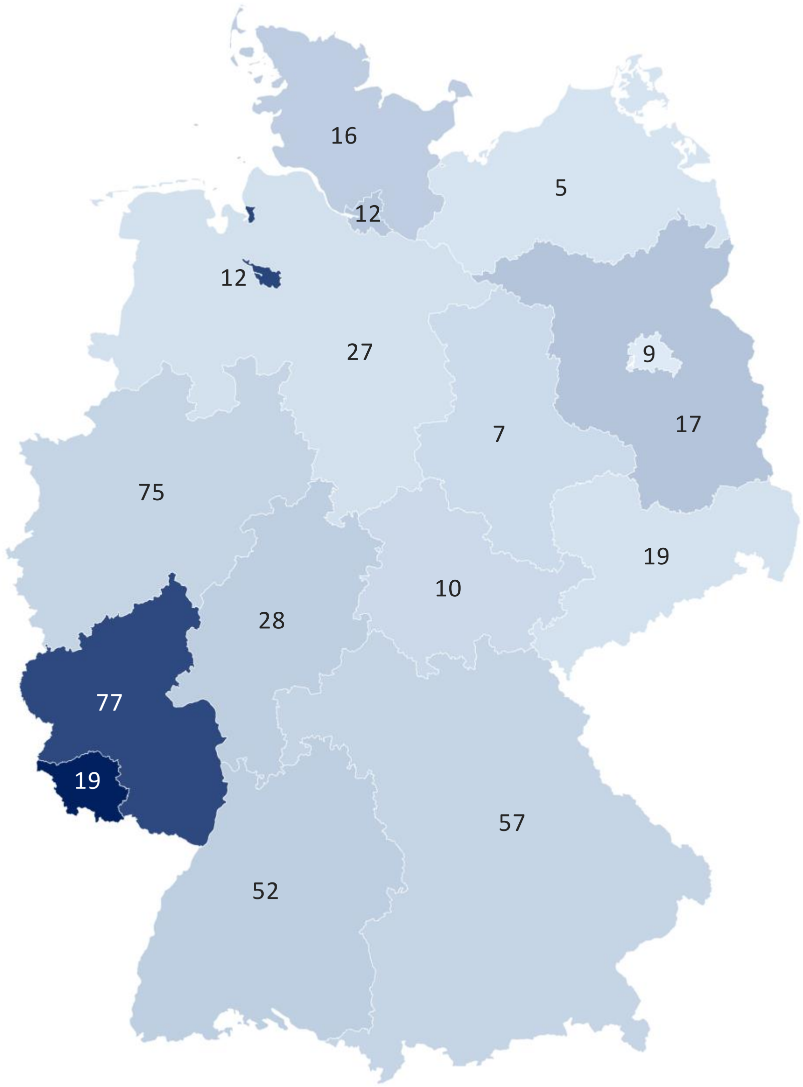


Number of respondents (n = 442) by federal states (n = 16) across Germany. The colours indicate the ratio between the number of responding physiotherapists and the number of employed physiotherapists subject to social security within each federal state, with dark and light blue shades indicating a higher and lower participation rate, respectively (Data source: PhysioDeutschland Deutscher Verband für Physiotherapie (ZVK) e. V. Zahlen, Daten und Fakten zur Physiotherapie. 2022).

Table A: Physiotherapy interventions for hip osteoarthritis (absolute numbers)

| | Always | Mostly | Sometimes | Never | No answer |
|---|---|---|---|---|---|
| Exercise therapy | 336 | 88 | 17 | 1 | 0 |
| Self-management advice | 306 | 107 | 26 | 3 | 0 |
| Pain management advice | 216 | 161 | 32 | 3 | 1 |
| Stress management | 69 | 152 | 161 | 30 | 1 |
| Education | 245 | 80 | 47 | 24 | 44 |
| Pain science | 217 | 93 | 12 | 2 | 1 |
| Exercise dose | 214 | 93 | 17 | 0 | 0 |
| Benefits of a healthy lifestyle | 185 | 108 | 29 | 2 | 1 |
| Pathology and risk factors | 170 | 114 | 36 | 2 | 3 |
| Impact on function, activity, participation | 158 | 125 | 40 | 2 | 0 |
| Joint protection strategies | 151 | 87 | 65 | 18 | 4 |
| Weight reduction | 100 | 106 | 113 | 4 | 2 |
| Manual therapy | 150 | 161 | 113 | 17 | 1 |
| Joint traction | 51 | 157 | 149 | 83 | 1 |
| Gait aids | 68 | 117 | 222 | 32 | 3 |
| Massage | 32 | 87 | 241 | 81 | 1 |
| Aquatic exercise | 15 | 66 | 120 | 237 | 3 |
| Thermotherapy (hot therapy) | 6 | 75 | 231 | 122 | 7 |
| Kinesio taping | 5 | 73 | 215 | 147 | 2 |
| Orthopaedic footwear (e.g. insoles) | 8 | 62 | 232 | 136 | 4 |
| Thermotherapy (cold therapy) | 5 | 52 | 220 | 157 | 7 |
| Ultrasound | 7 | 43 | 115 | 273 | 4 |
| TENS | 3 | 37 | 148 | 247 | 6 |
| Interferential current therapy | 4 | 27 | 124 | 281 | 6 |
| Braces/orthoses | 2 | 20 | 216 | 194 | 10 |
| Neuromuscular electric stimulation | 1 | 16 | 76 | 340 | 9 |
| Balneotherapy | 2 | 14 | 57 | 364 | 4 |
| Whole-body vibration exercise | 2 | 13 | 64 | 354 | 8 |
| Infrared therapy | 0 | 11 | 71 | 353 | 6 |
| Acupuncture | 0 | 11 | 51 | 368 | 12 |
| Electromagnetic field therapy | 1 | 10 | 28 | 395 | 8 |
| Shock wave therapy | 1 | 5 | 47 | 376 | 11 |
| Laser therapy | 0 | 4 | 24 | 403 | 11 |
| Short-wave diathermy | 2 | 1 | 28 | 395 | 16 |

*TENS* Transcutaneous electrical nerve stimulation

Table B: Physiotherapy interventions for knee osteoarthritis (absolute numbers)

| | Always | Mostly | Sometimes | Never | No Answer |
|---|---|---|---|---|---|
| Exercise therapy | 366 | 60 | 8 | 7 | 1 |
| Self-management advice | 289 | 106 | 40 | 5 | 2 |
| Pain management advice | 200 | 158 | 33 | 0 | 2 |
| Stress management | 76 | 133 | 155 | 30 | 0 |
| Education | 263 | 68 | 41 | 26 | 43 |
| Pain science | 203 | 105 | 23 | 0 | 0 |
| Exercise dose | 194 | 110 | 25 | 1 | 1 |
| Benefits of a healthy lifestyle | 183 | 116 | 29 | 2 | 1 |
| Impact on function, activity, participation | 170 | 126 | 31 | 4 | 0 |
| Pathology and risk factors | 183 | 106 | 40 | 2 | 0 |
| Joint protection strategies | 150 | 109 | 54 | 17 | 1 |
| Weight reduction | 99 | 123 | 99 | 7 | 3 |
| Manual therapy | 152 | 159 | 112 | 17 | 2 |
| Joint traction | 65 | 134 | 158 | 84 | 1 |
| Gait aids | 53 | 98 | 246 | 44 | 1 |
| Massage | 38 | 87 | 226 | 88 | 2 |
| Kinesio taping | 11 | 106 | 195 | 130 | 0 |
| Aquatic exercise | 20 | 66 | 107 | 241 | 7 |
| Thermotherapy (cold therapy) | 9 | 73 | 230 | 127 | 3 |
| Orthopaedic footwear (e.g. insoles) | 11 | 69 | 229 | 130 | 2 |
| Thermotherapy (hot therapy) | 8 | 72 | 227 | 128 | 6 |
| Braces/orthoses | 6 | 51 | 259 | 123 | 3 |
| Ultrasound | 6 | 47 | 119 | 268 | 2 |
| TENS | 5 | 42 | 130 | 260 | 5 |
| Interferential current therapy | 2 | 22 | 105 | 309 | 4 |
| Balneotherapy | 3 | 18 | 55 | 359 | 7 |
| Neuromuscular electric stimulation | 3 | 16 | 61 | 352 | 10 |
| Acupuncture | 1 | 15 | 77 | 345 | 4 |
| Whole-body vibration exercise | 1 | 15 | 58 | 361 | 7 |
| Infrared therapy | 0 | 12 | 50 | 376 | 4 |
| Electromagnetic field therapy | 2 | 10 | 30 | 394 | 6 |
| Shock wave therapy | 1 | 6 | 33 | 393 | 8 |
| Laser therapy | 0 | 6 | 15 | 410 | 9 |
| Short-wave diathermy | 0 | 3 | 23 | 408 | 7 |

*TENS* Transcutaneous electrical nerve stimulation

Table C: Determinants associated with choosing different treatment interventions for patients with hip OA

| | Exercise therapy | | Self-management advice | | Education | | Manual therapy | | Joint traction | |
|---|---|---|---|---|---|---|---|---|---|---|
| | Choosing n (%) | p-value | Choosing n (%) | p-value | Choosing n (%) | p-value | Choosing n (%) | p-value | Choosing n (%) | p-value |
| Age | | | | | | | | | | |
| 20-30 years | 113 (100) | 0.02 | 101 (89.4) | 0.08 | 94 (88.7) | 0.04 | 68 (60.2) | <0.000625* | 42 (37.2) | 0.03 |
| 31-40 years | 108 (94.7) | | 106 (93) | | 88 (83.8) | | 73 (64) | | 52 (46) | |
| ≥ 41 years | 201 (94.4) | | 204 (95.8) | | 142 (77.2) | | 168 (78.9) | | 112 (52.8) | |
| Gender | | | | | | | | | | |
| Female | 275 (95.5) | 0.72 | 273 (94.8) | 0.16 | 206 (81.7) | 0.96 | 213 (74) | 0.04 | 150 (52.4) | <0.01 |
| Male | 147 (96.7) | | 138 (90.8) | | 118 (82.5) | | 98 (64.5) | | 58 (38.2) | |
| Highest professional degree | | | | | | | | | | |
| Diploma (vocational school) | 285 (95.6) | 0.41 | 277 (93) | 0.62 | 201 (77) | <0.000625* | 217 (72.8) | 0.06 | 153 (51.3) | <0.01 |
| Bachelor's degree or higher | 131 (97.8) | | 127 (94.8) | | 120 (93.8) | | 85 (63.4) | | 49 (36.8) | |
| Work experience | | | | | | | | | | |
| 0-5 years | 109 (100) | 0.03 | 100 (91.7) | 0.08 | 95 (89.6) | 0.10 | 57 (52.3) | <0.000625* | 39 (35.8) | 0.02 |
| 6-15 years | 103 (95.4) | | 97 (89.8) | | 81 (81.8) | | 75 (70.1) | | 50 (46.7) | |
| 16-25 years | 95 (95.0) | | 98 (98.0) | | 64 (79.0) | | 74 (74.0) | | 49 (49.5) | |
| > 25 | 117 (93.6) | | 118 (94.4) | | 85 (77.3) | | 105 (84.0) | | 70 (56.0) | |
| Work setting | | | | | | | | | | |
| Private practice | 361 (95.5) | 0.75 | 351 (92.9) | 0.75 | 273 (81.3) | 0.36 | 270 (71.6) | 0.40 | 182 (48.4) | 0.62 |
| Hospital | 27 (96.4) | | 27 (96.4) | | 22 (81.5) | | 17 (60.7) | | 11 (39.3) | |
| Rehabilitation clinic | 29 (100) | | 28 (96.6) | | 25 (92.6) | | 22 (75.9) | | 13 (44.8) | |
| Size of city/municipality | | | | | | | | | | |
| < 20,000 inhabitants | 178 (94.7) | 0.29 | 175 (93.1) | 0.76 | 128 (77.6) | 0.07 | 147 (78.6) | <0.001 | 100 (54.5) | 0.02 |
| ≥ 20,000 inhabitants | 238 (97.1) | | 231 (94.3) | | 192 (85.3) | | 156 (63.7) | | 102 (41.8) | |
| Awareness of CPGs | | | | | | | | | | |
| Yes | 212 (100) | <0.000625* | 203 (95.8) | 0.19 | 185 (91.1) | <0.000625* | 131 (61.8) | <0.000625* | 82 (38.9) | <0.005 |
| No | 151 (92.6) | | 150 (92.0) | | 106 (73.6) | | 130 (79.8) | | 90 (55.2) | |
| Workload (OA patients/week) | | | | | | | | | | |
| ≤ 5 | 196 (95.1) | 0.60 | 192 (93.2) | 1 | 158 (85.4) | 0.16 | 130 (63.4) | <0.005 | 88 (43.1) | 0.62 |
| > 5 | 226 (96.6) | | 219 (93.6) | | 166 (79.4) | | 179 (76.5) | | 119 (50.9) | |

*CPG* Clinical practice guideline; *OA* Osteoarthritis; * Significant after Bonferroni correction ($p < 0.000625$)

Table D: Determinants associated with choosing different treatment interventions for patients with knee OA

| | Exercise therapy | | Self-management advice | | Education | | Manual therapy | | Joint traction | |
|---|---|---|---|---|---|---|---|---|---|---|
| | Choosing n (%) | p-value | Choosing n (%) | p-value | Choosing n (%) | p-value | Choosing n (%) | p-value | Choosing n (%) | p-value |
| Age | | | | | | | | | | |
| 20-30 years | 113 (100) | 0.02 | 95 (84.8) | 0.14 | 95 (89.6) | 0.04 | 69 (61.1) | <0.000625* | 40 (35.4) | 0.01 |
| 31-40 years | 111 (97.4) | | 103 (91.2) | | 89 (84.8) | | 70 (61.4) | | 48 (42.1) | |
| ≥ 41 years | 200 (94.3) | | 195 (91.5) | | 146 (78.5) | | 171 (80.7) | | 110 (51.9) | |
| Gender | | | | | | | | | | |
| Female | 278 (96.5) | 1 | 260 (90.9) | 0.34 | 210 (83.0) | 1 | 214 (74.8) | 0.01 | 149 (51.9) | <0.000625* |
| Male | 146 (96.7) | | 133 (87.5) | | 120 (83.3) | | 96 (63.1) | | 50 (32.8) | |
| Highest professional degree | | | | | | | | | | |
| Diploma (vocational school) | 287 (96.6) | 1 | 266 (89.6) | 0.78 | 207 (79.3) | <0.001 | 222 (74.7) | <0.01 | 146 (49.0) | 0.01 |
| Bachelor's degree or higher | 130 (97).0 | | 121 (91.0) | | 120 (92.3) | | 82 (61.2) | | 48 (35.8) | |
| Work experience | | | | | | | | | | |
| 0-5 years | 108 (99.1) | 0.05 | 93 (86.1) | 0.38 | 97 (91.5) | 0.05 | 59 (54.1) | <0.000625* | 38 (34.9) | 0.01 |
| 6-15 years | 106 (98.1) | | 95 (88.8) | | 81 (82.7) | | 72 (68.6) | | 44 (41.1) | |
| 16-25 years | 97 (97.0) | | 91 (91.0) | | 65 (78.3) | | 76 (76.0) | | 48 (48.0) | |
| > 25 | 115 (92.7) | | 116 (92.8) | | 88 (87.0) | | 104 (83.9) | | 69 (55.2) | |
| Work setting | | | | | | | | | | |
| Private practice | 365 (96.8) | 0.38 | 337 (89.4) | 0.82 | 279 (82.3) | 0.46 | 270 (71.8) | 0.16 | 173 (45.9) | 0.73 |
| Hospital | 27 (96.4) | | 26 (92.9) | | 22 (84.6) | | 16 (57.1) | | 11 (39.3) | |
| Rehabilitation clinic | 27 (93.1) | | 26 (92.9) | | 25 (92.6) | | 23 (79.3) | | 12 (41.4) | |
| Size of city/municipality | | | | | | | | | | |
| < 20,000 inhabitants | 184 (97.9) | 0.38 | 166 (89.2) | 0.76 | 133 (75.1) | 0.14 | 148 (79.1) | <0.001 | 97 (51.8) | 0.01 |
| ≥ 20,000 inhabitants | 234 (95.9) | | 222 (90.6) | | 193 (85.8) | | 156 (63.7) | | 96 (39.2) | |
| Awareness of CPGs | | | | | | | | | | |
| Yes | 209 (98.6) | 0.06 | 194 (92.4) | 0.06 | 188 (92.6) | <0.000625* | 132 (62.3) | <0.000625* | 80 (37.7) | 0.03 |
| No | 154 (95.1) | | 140 (85.9) | | 108 (74.5) | | 130 (79.8) | | 81 (49.7) | |
| Workload (OA patients/week) | | | | | | | | | | |
| ≤ 5 | 200 (97.1) | 0.78 | 180 (87.4) | 0.17 | 163 (86.2) | 0.22 | 134 (65.7) | 0.05 | 83 (40.5) | 0.10 |
| > 5 | 224 (96.1) | | 213 (91.8) | | 168 (81.2) | | 175 (74.8) | | 114 (48.7) | |

*CPG* Clinical practice guideline; *OA* Osteoarthritis; * Significant after Bonferroni correction (p < 0.000625)

Table E: Frequency of the combined use of different treatments for patients with hip and knee OA

| exercise therapy | self-management advice | education | manual therapy | joint traction | gait aids | massage | aquatic exercise | Hip OA (n (%)) | Knee OA (n (%)) |
|---|---|---|---|---|---|---|---|---|---|
| ● | ● | ● | | | | | | 58 (13.1) | 60 (13.6) |
| ● | ● | ● | ● | | | | | 20 (4.5) | 21 (4.8) |
| ● | ● | ● | | | ● | | | 16 (3.6) | 11 (2.5) |
| ● | ● | ● | ● | ● | | | | 11 (2.5) | 14 (3.2) |
| ● | ● | ● | ● | | ● | | | 13 (2.9) | 6 (1.4) |
| ● | ● | ● | | | | | ● | 8 (1.8) | 10 (2.3) |
| ● | ● | ● | ● | ● | | ● | | 6 (1.4) | 7 (1.6) |
| ● | ● | ● | ● | ● | ● | | | 6 (1.4) | 5 (1.1) |
| ● | ● | | ● | ● | | | | 5 (1.1) | 5 (1.1) |
| ● | ● | ● | ● | ● | ● | ● | | 9 (2.0) | |
| ● | ● | | ● | | | | | | 7 (1.6) |
| ● | ● | ● | ● | | | ● | | | 5 (1.1) |

*OA* Osteoarthritis. Note: Combinations of treatment modalities used by < 1% of respondents are not displayed here.

Figure B: Factors influencing the choice of treatment (n = 438)

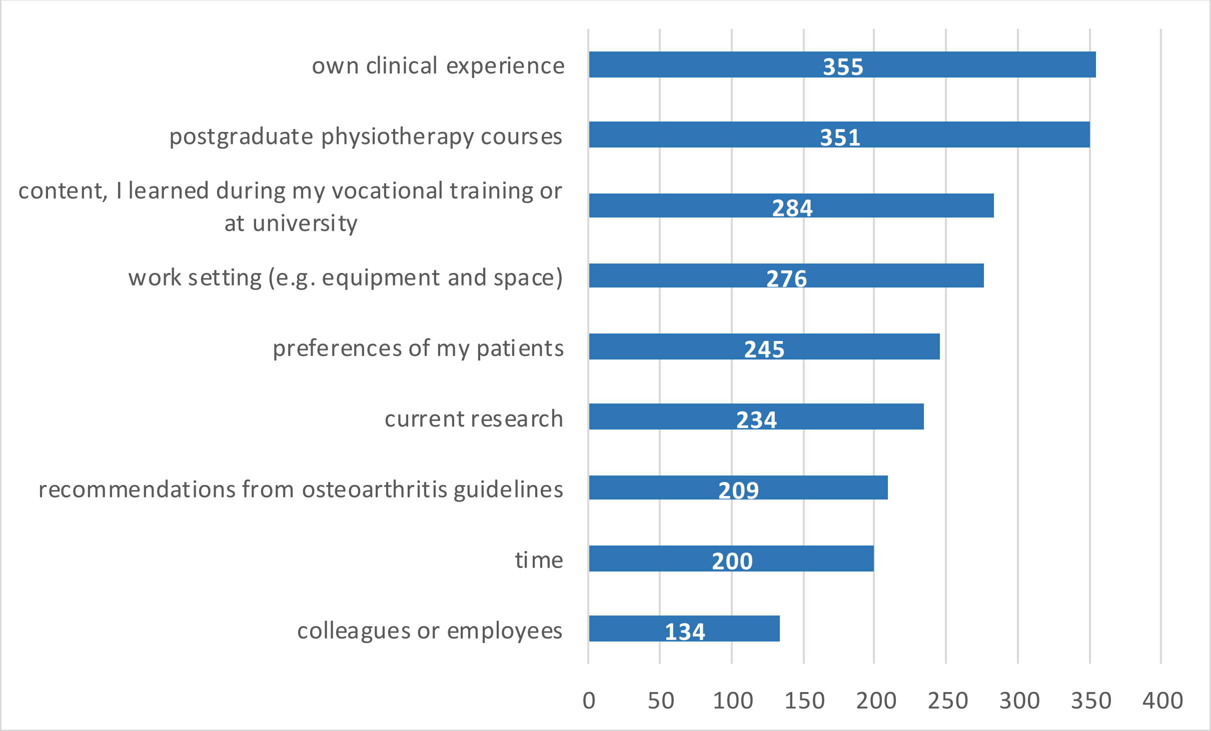

Table F: Perceived barriers and facilitators specific to the AWMF guidelines for hip and knee OA

| | Fully disagree | Disagree | Do not agree or disagree | Agree | Fully agree | No answer | Missing |
|---|---|---|---|---|---|---|---|
| | n (%) | | | | | | |
| The guideline leaves enough room for me to make my own conclusions. | 0 (0) | 8 (5.6) | 6 (4.2) | 99 (69.2) | 21 (14.7) | 8 (5.6) | 1 (0.7) |
| The guideline leaves enough room to weight the wishes of the patient. | 0 (0) | 12 (8.4) | 13 (9.1) | 91 (63.6) | 18 (12.6) | 8 (5.6) | 1 (0.7) |
| The guideline is a good starting point for my self-study. | 0 (0) | 5 (3.5) | 17 (11.9) | 85 (59.4) | 34 (23.8) | 2 (1.4) | 0 (0) |
| I did not thoroughly read nor remember the guideline. | 9 (6.3) | 67 (46.9) | 40 (28.0) | 21 (14.7) | 2 (1.4) | 4 (2.8) | 0 (0) |
| I wish to know more about the guideline before I decide to apply it. | 12 (8.4) | 58 (40.6) | 34 (23.8) | 23 (16.1) | 5 (3.5) | 11 (7.7) | 0 (0) |
| I have problems changing my old routines. | 24 (16.8) | 81 (56.6) | 14 (9.8) | 19 (13.3) | 1 (0.7) | 3 (2.1) | 1 (0.7) |
| I think parts of the guideline are incorrect. | 12 (8.4) | 43 (30.1) | 40 (28.0) | 21 (14.7) | 4 (2.8) | 22 (15.5) | 1 (0.7) |
| I have a general resistance to working according protocols. | 69 (48.3) | 42 (29.4) | 22 (15.4) | 4 (2.8) | 4 (2.8) | 2 (1.4) | 0 (0) |
| My colleagues do not cooperate in applying the guideline. | 12 (8.4) | 21 (14.7) | 32 (22.4) | 46 (32.2) | 12 (8.4) | 19 (13.3) | 1 (0.7) |
| Other health care professionals do not cooperate in applying the guideline. | 11 (7.7) | 11 (7.7) | 21 (14.7) | 60 (42.0) | 15 (10.5) | 24 (16.8) | 1 (0.7) |
| Managers/directors do not cooperate in applying the guideline. | 19 (13.3) | 29 (20.3) | 26 (18.2) | 29 (20.3) | 11 (7.7) | 28 (19.6) | 1 (0.7) |
| Patients do not cooperate applying the guideline. | 9 (6.3) | 40 (28.0) | 38 (26.6) | 41 (28.7) | 5 (3.5) | 8 (5.6) | 2 (1.4) |
| Working to the guideline is too time consuming. | 8 (5.6) | 60 (42.0) | 28 (19.6) | 33 (23.1) | 6 (4.2) | 8 (5.6) | 0 (0) |
| The guideline does not fit into my ways of working in practice. | 28 (19.6) | 62 (43.4) | 28 (19.6) | 17 (11.9) | 2 (1.4) | 5 (3.5) | 1 (0.7) |
| Working according to this guideline requires financial compensation. | 15 (10.5) | 51 (35.7) | 15 (10.5) | 38 (26.6) | 9 (6.3) | 14 (9.8) | 1 (0.7) |
| The layout of the guideline makes it handy for use. | 4 (2.8) | 17 (11.9) | 42 (29.4) | 51 (35.7) | 1 (0.7) | 27 (18.9) | 1 (0.7) |
