# Supplementary material for: Physiotherapy for patients with hip and knee osteoarthritis in Germany: a survey of current practice
Source: BMC Musculoskelet Disord. 2023 May 26;24:424. doi: 10.1186/s12891-023-06464-0 (PMC10262543; doi:10.1186/s12891-023-06464-0)
Supplement: Supplementary file 3 — Supplementary Material 3 [file 12891_2023_6464_MOESM3_ESM.pdf]

Physiotherapeutische Versorgungssituation von Patient*innen mit Hüft- und/oder Kniearthrose in Deutschland

Sehr geehrte Teilnehmerin, sehr geehrter Teilnehmer,

vielen Dank, dass Sie sich für unsere Online-Befragung zum Thema „Physiotherapeutische Versorgungssituation von Patient*innen mit Hüft- und/oder Kniearthrose in Deutschland“ interessieren.

Was ist das Ziel der Studie?
Ziel der Studie ist die Erfassung der aktuellen Versorgungssituation im physiotherapeutischen Kontext von Patient*innen mit Hüft- und/oder Kniearthrose in Deutschland. Wir möchten überprüfen, inwieweit die ergriffenen Maßnahmen den Empfehlungen der AWMF-Leitlinien Koxarthrose und Gonarthrose entsprechen und erfahren, welche Faktoren die Umsetzung der Leitlinienempfehlungen beeinflussen.

Was wird untersucht?
Wir würden Ihnen gerne einige Fragen zu den folgenden Themenbereichen stellen:

1. Allgemeine demografische und berufliche Angaben
2. Physiotherapeutische Versorgung von Patient*innen mit Hüft- und/oder Kniearthrose
3. Förderfaktoren und Barrieren in der Anwendung von Leitlinien

Wie zeitaufwändig ist die Teilnahme?
Die Teilnahme an der Online-Befragung nimmt ca. 10 Minuten in Anspruch.

Was passiert mit meinen Daten?
Die erhobenen Daten werden ausschließlich zu wissenschaftlichen Zwecken genutzt. Eine kommerzielle Nutzung ist ausgeschlossen. Die Auswertung der Daten geschieht in anonymisierter Form, d. h., ohne dass die Daten einer spezifischen Person zugeordnet werden können. Die vollständig anonymisierten Daten dieser Studie werden für mindestens zehn Jahre auf dem Server der Brandenburgischen Technischen Universität Cottbus-Senftenberg gespeichert und der Forschungsgemeinschaft in einem sicheren, internetbasierten Datenarchiv (Open Science Framework) zugänglich gemacht. Damit folgt diese Studie den Empfehlungen der Deutschen Forschungsgemeinschaft (DFG) zur Qualitätssicherung in der Forschung.

Welche Risiken gibt es?
Die Befragung ist mit keinen Risiken verbunden.

Ihre Teilnahme an dieser Studie erfolgt freiwillig. Sie können jederzeit ohne Angabe von Gründen Ihre Teilnahme zurückziehen, ohne dass Ihnen dadurch Nachteile entstehen. Sie sind nicht dazu verpflichtet jede Frage zu beantworten und können die Befragung jederzeit beenden.
Bitte wenden Sie sich an den Studienleiter, Herrn Prof. Dr. Christian Kopkow, falls Sie weitere Fragen zur Studie haben oder etwas an dieser Teilnehmerinformation nicht verstanden haben.

Prof. Dr. Christian Kopkow können Sie erreichen unter:
Telefon: 03573 - 85 741
Email: christian.kopkow@b-tu.de

Datenschutzerklärung

- *Ich willige ein, dass im Rahmen der Studie erhobene Daten auf elektronischen Datenträgern aufgezeichnet und in anonymisierter Form bei dem Erhebungs- und Auswertungszentrum* der Studie zur wissenschaftlichen Auswertung gespeichert sowie auf einem sicheren, internetbasierten Datenarchiv (Open Science Framework), den Empfehlungen der Deutschen Forschungsgemeinschaft (DFG) zur Qualitätssicherung in der Forschung folgend, zugänglich gemacht werden.*
- *Außerdem willige ich ein, dass ein autorisierter und zur Verschwiegenheit verpflichteter Beauftragter der Ethik-Kommission in meine beim Erhebungs- und Auswertungszentrum* vorhandenen personenbezogenen Daten Einsicht nehmen darf, soweit dies für die Überprüfung der Studie notwendig ist. Für diese Maßnahme entbinde ich das Erhebungs- und Auswertungszentrum von der Schweigepflicht.*
- *Ich weiß, dass ich meine Zustimmung jederzeit widerrufen kann, ohne dass mir daraus Nachteile entstehen.*

** Prof. Dr. Christian Kopkow, Brandenburgische Technische Universität Cottbus - Senftenberg, Fakultät 4 - Institut für Gesundheit, Fachgebiet Therapiewissenschaft I, Universitätsplatz 1, 01968 Senftenberg*

Ich habe die Teilnehmerinformation zur Studie „Physiotherapeutische Versorgungssituation von Patient*innen mit Hüft- und/oder Kniearthrose in Deutschland“ gelesen und verstanden. Ich erkläre hiermit meine Einwilligung zur Teilnahme an der genannten Studie und bin mit der Aufzeichnung meiner Daten und deren Weitergabe sowie der Einsichtnahme in meine personenbezogenen Daten in der oben beschriebenen Form einverstanden.

Sie müssen auf **„Weiter“** klicken, um an der Umfrage teilnehmen zu können.

Weiter

Bitte beantworten Sie folgende Fragen, um Ihre Teilnahmeberechtigung an dieser Studie zu prüfen:

1. *Arbeiten Sie aktuell als Physiotherapeut*in in Deutschland?

- Ja
- Nein

2. *Umfasst Ihre aktuelle Tätigkeit die Versorgung von Patient*innen mit Hüft- und/oder Kniearthrose?

- Ja
- Nein

Zurück | Weiter

3. Wie alt sind Sie?

Bitte auswählen..

4. Welches Geschlecht haben Sie?

- männlich
- weiblich
- divers
- Keine Antwort

5. In welchem Bundesland arbeiten Sie?

Bitte auswählen..

6. Wie viele Einwohner*innen leben in der Stadt (oder der Gemeinde), in welcher sich Ihr Arbeitsplatz befindet?

Wenn Sie mehrere Arbeitsverhältnisse haben, dann beantworten Sie diese Frage bitte in Bezug auf das Arbeitsverhältnis mit dem höchsten Umfang.

- < 5.000 Einwohner*innen (Landgemeinde)
- 5.000-20.000 Einwohner*innen (Kleinstadt)
- 20.000-100.000 Einwohner*innen (Mittelstadt)
- > 100.000 Einwohner*innen (Großstadt)
- Keine Antwort

7. Wie viele Jahre Berufserfahrung haben Sie als Physiotherapeut*in?

(gerundet auf volle Jahre nach "Berufsausbildung")

Bitte auswählen..

8. Wie viele Stunden pro Woche sind Sie aktuell durchschnittlich als Physiotherapeut*in tätig?

Bitte auswählen..

9.

Was ist Ihr derzeitiges Arbeitsumfeld?

Wenn Sie mehrere Arbeitsplätze haben, dann beantworten Sie diese Frage bitte in Bezug auf den Arbeitsplatz, an dem Sie am meisten tätig sind.

- freie Praxis
- Krankenhaus
- Rehabilitationsklinik
- Sonstiges:
- Keine Antwort

10.

Welcher ist Ihr höchster Abschluss, den Sie im Fachbereich Physiotherapie und/oder vergleichbarem Bereich (z. B. Gesundheitswissenschaften, Public Health, Sportwissenschaften) erworben haben?

- Staatsexamen
- Diplom
- Bachelor
- Master
- Promotion
- Sonstiges:
- Keine Antwort

11.

Wie viele Patient*innen behandeln Sie aktuell in einer typischen Arbeitswoche aufgrund ihrer Beschwerden durch Hüft- und/oder Kniearthrose (Einzel- und Gruppentherapie)?

Patient*innen, die mehrfach pro Woche behandelt werden, bitte nur einfach zählen.

Bitte auswählen..

Zurück

Weiter

Im Folgenden werden Sie zu Maßnahmen in der Behandlung von Patient*innen mit Hüft- und/oder Kniearthrose befragt.

Bitte beachten Sie, dass es sich hierbei um **zwei separate Tabellen** handelt. Die erste Tabelle bezieht sich dabei ausdrücklich nur auf die Behandlung von Hüftarthrose, in der zweiten Tabelle werden anschließend die Maßnahmen zur Behandlung von Kniearthrose erfragt.

Zurück

Weiter

12.

Bitte geben Sie an, wie häufig Sie folgende Maßnahmen in der Versorgung von Patient*innen mit **Hüftarthrose** anwenden bzw. empfehlen.

Die Maßnahmen sind in alphabetischer Reihenfolge angeordnet.

	Nie	Manchmal	Oft	Immer	Keine Antwort
Akupunktur	○	○	○	○	○
Balneotherapie (z. B. Thermal-/Mineralbäder)	○	○	○	○	○
Bewegungstherapie (z. B. Krafttraining, Ausdauertraining, funktionelle Übungen, Gleichgewichtstraining)	○	○	○	○	○
Edukation	○	○	○	●	○
► *zur Pathologie/Risikofaktoren von Arthrose*	○	○	○	○	○
► *zu Folgen der Erkrankung für die Leistungsfähigkeit in Bezug auf Funktion, Aktivität und Teilhabe*	○	○	○	○	○
► *zu gelenkschonenden Verhaltensweisen*	○	○	○	○	○
► *zur Bedeutung von Gewichtsreduktion*	○	○	○	○	○
► *zur Bedeutung eines gesunden Lebensstils (körperliche Aktivität, Ernährung)*	○	○	○	○	○
► *zu Schmerzen*	○	○	○	○	○
► *zur Belastungsdosierung*	○	○	○	○	○
Instruktionen zum Selbstmanagement	○	○	○	●	○
► *Strategien zum Umgang mit Schmerzen*	○	○	○	○	○
► *Stressbewältigung und Entspannung*	○	○	○	○	○
Gehhilfen (z. B. Gehstützen, Stöcke)	○	○	○	○	○
(Ganzkörper-) Vibrationstherapie	○	○	○	○	○
Hydrotherapie (Bewegungsbad)	○	○	○	○	○
Infrarottherapie	○	○	○	○	○
Interferenzstrom	○	○	○	○	○
(Kinesio-)Taping	○	○	○	○	○

Kurzwellentherapie	○	○	○	○	○
Lasertherapie (Low-Level-Lasertherapie, LLLT)	○	○	○	○	○
Magnetfeldtherapie	○	○	○	○	○
Manuelle Therapie (z. B. Gelenkmobilisation)	○	○	○	○	○
Massage	○	○	○	○	○
Neuromuskuläre elektrische Stimulation (NMES)	○	○	○	○	○
Orthesen/Bandagen	○	○	○	○	○
Orthopädietechnische Schuhversorgung (z. B. Einlagen, Erhöhung der Schuhsohlen)	○	○	○	○	○
Stoßwellentherapie	○	○	○	○	○
Thermotherapie (Kälteanwendungen)	○	○	○	○	○
Thermotherapie (Wärmeanwendungen)	○	○	○	○	○
Traktionsbehandlung (manuell oder mit Gerät)	○	○	○	○	○
Transkutane elektrische Nervenstimulation (TENS)	○	○	○	○	○
Ultraschalltherapie	○	○	○	○	○

Zurück

Weiter

13.

Bitte geben Sie an, wie häufig Sie folgende Maßnahmen in der Versorgung von Patient*innen mit **Kniearthrose** anwenden bzw. empfehlen.

Die Maßnahmen sind in alphabetischer Reihenfolge angeordnet.

	Nie	Manchmal	Oft	Immer	Keine Antwort
Akupunktur	○	○	○	○	○
Balneotherapie (z. B. Thermal-/Mineralbäder)	○	○	○	○	○
Bewegungstherapie (z. B. Krafttraining, Ausdauertraining, funktionelle Übungen, Gleichgewichtstraining)	○	○	○	○	○
Edukation	○	○	○	●	○
► *zur Pathologie/Risikofaktoren von Arthrose*	○	○	○	○	○
► *zu Folgen der Erkrankung für die Leistungsfähigkeit in Bezug auf Funktion, Aktivität und Teilhabe*	○	○	○	○	○
► *zu gelenkschonenden Verhaltensweisen*	○	○	○	○	○
► *zur Bedeutung von Gewichtsreduktion*	○	○	○	○	○
► *zur Bedeutung eines gesunden Lebensstils (körperliche Aktivität, Ernährung)*	○	○	○	○	○
► *zu Schmerzen*	○	○	○	○	○
► *zur Belastungsdosierung*	○	○	○	○	○
Instruktionen zum Selbstmanagement	○	○	○	●	○
► *Strategien zum Umgang mit Schmerzen*	○	○	○	○	○
► *Stressbewältigung und Entspannung*	○	○	○	○	○
Gehhilfen (z. B. Gehstützen, Stöcke)	○	○	○	○	○
(Ganzkörper-)Vibrationstherapie	○	○	○	○	○
Hydrotherapie (Bewegungsbad)	○	○	○	○	○
Infrarottherapie	○	○	○	○	○
Interferenzstrom	○	○	○	○	○
(Kinesio-)Taping	○	○	○	○	○

Kurzwellentherapie	○	○	○	○	○
Lasertherapie (Low-Level-Lasertherapie, LLLT)	○	○	○	○	○
Magnetfeldtherapie	○	○	○	○	○
Manuelle Therapie (z. B. Gelenkmobilisation)	○	○	○	○	○
Massage	○	○	○	○	○
Neuromuskuläre elektrische Stimulation (NMES)	○	○	○	○	○
Orthesen/Bandagen	○	○	○	○	○
Orthopädietechnische Schuhversorgung (z. B. Einlagen, Erhöhung der Schuhsohlen)	○	○	○	○	○
Stoßwellentherapie	○	○	○	○	○
Thermotherapie (Kälteanwendungen)	○	○	○	○	○
Thermotherapie (Wärmeanwendungen)	○	○	○	○	○
Traktionsbehandlung (manuell oder mit Gerät)	○	○	○	○	○
Transkutane elektrische Nervenstimulation (TENS)	○	○	○	○	○
Ultraschalltherapie	○	○	○	○	○

Zurück

Weiter

14.

Hinsichtlich Ihrer Antworten auf die vorherigen Fragen - welche Faktoren beeinflussen die Wahl der Behandlungs-maßnahmen, die Sie anbieten oder vorschlagen würden?

Mehrfachnennungen möglich

- [ ] eigene klinische Erfahrung
- [ ] Arbeitsumfeld/Arbeitsplatz (z. B. Geräte und Räumlichkeiten)
- [ ] Fort- und Weiterbildungen
- [ ] Empfehlungen aus Leitlinien für Arthrose
- [ ] Inhalte, die ich während der Ausbildung/des Studiums gelernt habe
- [ ] meine Kolleg*innen oder Mitarbeiter*innen
- [ ] Wünsche und Vorstellungen der Patient*innen
- [ ] zur Verfügung stehende Zeit
- [ ] aktuelle wissenschaftliche Studien
- [ ] Sonstiges: ______
- [ ] Keine Antwort

Zurück

Weiter

15. Sind Ihnen Leitlinien zur Versorgung von Patient*innen mit Hüft- und/oder Kniearthrose bekannt?

- Ja
- Nein
- Keine Antwort

Question 16 only appears if question 15 was answered with "Ja".

16. Welche der folgenden Leitlinien sind Ihnen bekannt?

Mehrfachnennungen möglich

- **AAOS** - Clinical Practice Guideline on the Management of Osteoarthritis of the Hip
- **AAOS** - Clinical Practice Guideline on Treatment of Osteoarthritis of the Knee
- **ACR** - Guideline for the Management of Osteoarthritis of the Hand, Hip, and Knee
- **APTA** - Hip Pain and Mobility Deficits - Hip Osteoarthritis: Revision 2017
- **AWMF** - S2k-Leitlinie „Gonarthrose"
- **AWMF** - S2k-Leitlinie „Koxarthrose"
- **KNGF** - Guideline for Physical Therapy in patients with Osteoarthritis of the hip and knee
- **EULAR** - Recommendations for the non-pharmacological core management of hip and knee osteoarthritis
- **NICE** - Osteoarthritis: Care and Management in Adults
- **OARSI** - Guidelines for the non-surgical management of knee, hip and polyarticular osteoarthritis
- **RACGP** - Guideline for the management of knee and hip osteoarthritis
- Keine Antwort

Question 17 only appears if question 15 was answered with „Ja“.

17. Woher kennen Sie diese Leitlinien?

Mehrfachnennungen möglich

- [ ] Ausbildung/Studium
- [ ] Fort-/Weiterbildung
- [ ] Kolleg*innen/Mitarbeiter*innen
- [ ] Kongresse
- [ ] Webinare/Podcasts
- [ ] Soziale Medien wie Facebook, Twitter, Instagram, Youtube
- [ ] Online (Google, Websites für Leitlinien)
- [ ] Fachzeitschriften für Medizin/Gesundheit/Physiotherapie
- [ ] Sonstiges: ______
- [ ] Keine Antwort

Zurück Weiter

Question 18 only appears if question 16 (screen 8a) was answered with “AWMF - S2k-Leitlinie Gonarthrose” and/or “AWMF - S2k-Leitlinie Koxarthrose”.

18.

Sie haben angegeben die **AWMF S2k-Leitlinie „Koxarthrose“ und/oder "Gonarthrose"** zu kennen.
Im Folgenden finden Sie eine Reihe von Aussagen über das Arbeiten nach dieser Leitlinie. Wir bitten Sie, anzugeben, inwieweit Sie der jeweiligen Aussage zustimmen oder nicht zustimmen. Falls Sie keine eindeutige Meinung diesbezüglich haben, bitten wir Sie eine Tendenz in Richtung „stimme zu“ oder „stimme nicht zu“ anzugeben. Falls Sie trotzdem unentschlossen sind, haben Sie die Möglichkeit „weder noch“ anzukreuzen.

	Stimme überhaupt nicht zu	Stimme nicht zu	Weder noch	Stimme zu	Stimme vollständig zu	Keine Angabe
Die Leitlinie lässt mir genügend Freiraum für eigene Entscheidungen.						
Die Leitlinie lässt mir genügend Freiraum, um die Wünsche der Patient*innen zu berücksichtigen.						
Die Leitlinie ist ein guter Ausgangspunkt, um mich weiterführend mit dem Thema auseinanderzusetzen.						
Ich habe die Leitlinie nicht gründlich gelesen oder sie mir nicht gemerkt.						
Ich würde gerne mehr über die Leitlinie wissen, bevor ich mich dazu entschließe diese zu benutzen.						
Ich habe Schwierigkeiten damit, meine gewohnten Abläufe zu ändern.						
Ich denke, dass bestimmte Abschnitte der Leitlinie nicht richtig sind.						
Ich bin generell dagegen, nach Behandlungsstandards zu arbeiten.						
Meine Kolleg*innen arbeiten an der **Umsetzung** der Leitlinie nicht mit.						
Andere, an der Versorgung beteiligte Personen, arbeiten an der **Umsetzung** der Leitlinie nicht mit.						
Meine Vorgesetzten arbeiten an der **Umsetzung** der Leitlinie nicht mit.						
Patient*innen arbeiten an der **Umsetzung** der Leitlinie nicht mit.						
Das Arbeiten nach der Leitlinie nimmt viel Zeit in Anspruch.						
Die Leitlinie passt nicht gut zur Arbeitsweise in meiner Einrichtung/meinem Bereich bzw. zu meinem Arbeitsstil.						

Das Arbeiten nach dieser Leitlinie erfordert einen finanziellen Ausgleich.	○	○	○	○	○	○
Das Layout dieser Leitlinie erleichtert die Nutzung.	○	○	○	○	○	○

Zurück

Weiter

Question 19 only appears if question 15 (screen 8a) was answered with „Ja“.

19. Wie häufig nutzen Sie Leitlinien in Ihrer Versorgung von Patient*innen mit Hüft- und/oder Kniearthrose?

- ○ Immer
- ○ Oft
- ○ Manchmal
- ○ Nie
- ○ Keine Antwort

Question 20 only appears if question 19 was answered with "Manchmal" or "Nie".

20. Aus welchen Gründen?

Mehrfachnennungen möglich

- ☐ Ich habe keine Zeit, Leitlinien zu lesen
- ☐ Ich habe keine Zeit, die Empfehlungen aus Leitlinien am Arbeitsplatz umzusetzen
- ☐ Ich habe kein Interesse daran, die Empfehlungen aus Leitlinien umzusetzen
- ☐ Ich weiß nicht, wie und wo ich Leitlinien finden kann
- ☐ Es gibt keine/zu wenig Leitlinien
- ☐ Die Empfehlungen aus Leitlinien sind zu unspezifisch und werden der Individualität meiner Patient*innen nicht gerecht
- ☐ Empfehlungen aus Leitlinien sind nicht hilfreich, um die Patient*innenversorgung zu verbessern
- ☐ Der Einsatz von Leitlinien wird von meinen Arbeitskolleg*innen nicht unterstützt
- ☐ Der Einsatz von Leitlinien wird von meinem Arbeitgeber nicht unterstützt
- ☐ Empfehlungen aus Leitlinien widersprechen meiner klinischen Expertise
- ☐ Empfehlungen aus Leitlinien behindern mich in meiner klinischen Entscheidungsfindung
- ☐ Die Wünsche meiner Patient*innen stimmen nicht mit den Empfehlungen aus Leitlinien überein
- ☐ Arthrose der Hüft-/Kniegelenke ist keine ernste Erkrankung und bedarf keiner Versorgung nach Leitlinien
- ☐ Ich habe Schwierigkeiten, Leitlinien zu verstehen und kritisch zu bewerten
- ☐ Ich habe nicht die nötigen Ressourcen (z. B. Platz, Ausrüstung), um Empfehlungen aus Leitlinien in der klinischen Praxis umzusetzen
- ☐ Sonstiges: ________
- ☐ Keine Antwort

Zurück | Weiter

15.

Sind Ihnen Leitlinien zur Versorgung von Patient*innen mit Hüft- und/oder Kniearthrose bekannt?

- Ja
- Nein
- Keine Antwort

Question 21 only appears if question 15 was answered with “Nein”.

21.

Haben Sie generelles Interesse daran, Empfehlungen aus Leitlinien in Ihre tägliche Arbeit mit Patient*innen mit Hüft- und/oder Kniearthrose einzubeziehen?

- Ja
- Nein
- Keine Antwort

Zurück

Weiter

Question 22 only appears if question 15 (screen 8b) was answered with „Nein“.

22. Wie häufig nutzen Sie generell Leitlinien in der Versorgung Ihrer Patient*innen?

- ○ Immer
- ○ Oft
- ○ Manchmal
- ○ Nie
- ○ Keine Antwort

Question 23 only appears if question 22 was answered with “Manchmal” or “Nie”.

23. Aus welchen Gründen?

Mehrfachnennungen möglich

- ☐ Ich kenne keine Leitlinien
- ☐ Ich habe keine Zeit, Leitlinien zu lesen
- ☐ Ich habe keine Zeit, die Empfehlungen aus Leitlinien am Arbeitsplatz umzusetzen
- ☐ Ich habe kein Interesse daran, die Empfehlungen aus Leitlinien umzusetzen
- ☐ Ich weiß nicht, wie und wo ich Leitlinien finden kann
- ☐ Es gibt keine/zu wenige Leitlinien
- ☐ Die Empfehlungen aus Leitlinien sind zu unspezifisch und werden der Individualität meiner Patient*innen nicht gerecht
- ☐ Empfehlungen aus Leitlinien sind nicht hilfreich, um die Patient*innenversorgung zu verbessern
- ☐ Der Einsatz von Leitlinien wird von meinen Arbeitskolleg*innen nicht unterstützt
- ☐ Der Einsatz von Leitlinien wird von meinem Arbeitgeber nicht unterstützt
- ☐ Empfehlungen von Leitlinien widersprechen meiner klinischen Expertise
- ☐ Empfehlungen aus Leitlinien behindern mich in meiner klinischen Entscheidungsfindung
- ☐ Die Wünsche meiner Patient*innen stimmen nicht mit den Empfehlungen aus Leitlinien überein
- ☐ Ich habe Schwierigkeiten, Leitlinien zu verstehen und kritisch zu bewerten
- ☐ Ich habe nicht die nötigen Ressourcen (z. B. Platz, Ausrüstung), um Empfehlungen aus Leitlinien in der klinischen Praxis umzusetzen
- ☐ Sonstiges: ________
- ☐ Keine Antwort

Zurück Weiter

Vielen Dank für Ihre Teilnahme!

Wir möchten uns ganz herzlich für Ihre Mithilfe bedanken.

Ihre Antworten wurden gespeichert. Sie können das Browser-Fenster nun schließen.

Screen 11b only appears if question 1 or 2 was answered with “Nein”.

Vielen Dank für Ihr Interesse an unserer Studie!

Leider erfüllen Sie nicht alle Voraussetzungen für die Teilnahme an der Befragung.

Zurück

Absenden
